# The influence of diet and environment on the gut microbial community of field crickets

**DOI:** 10.1002/ece3.3977

**Published:** 2018-04-16

**Authors:** Soon Hwee Ng, Michael Stat, Michael Bunce, Leigh W. Simmons

**Affiliations:** ^1^ Centre for Evolutionary Biology School of Biological Sciences University of Western Australia Crawley Australia; ^2^ Department of Biological Sciences Macquarie University Sydney Australia; ^3^ Trace and Environmental DNA (TrEnD) Laboratory Department of Environment and Agriculture Curtin University Perth Australia

**Keywords:** community membership, community structure, diet, gut microbial diversity, predictive metagenomes, *Teleogryllus oceanicus*

## Abstract

The extent to which diet and environment influence gut community membership (presence or absence of taxa) and structure (individual taxon abundance) is the subject of growing interest in microbiome research. Here, we examined the gut bacterial communities of three cricket groups: (1) wild caught field crickets, (2) laboratory‐reared crickets fed cat chow, and (3) laboratory‐reared crickets fed chemically defined diets. We found that both environment and diet greatly altered the structure of the gut bacterial community. Wild crickets had greater gut microbial diversity and higher *Firmicutes* to *Bacteroidetes* ratios, in contrast to laboratory‐reared crickets. Predictive metagenomes revealed that laboratory‐reared crickets were significantly enriched in amino acid degradation pathways, while wild crickets had a higher relative abundance of peptidases that would aid in amino acid release. Although wild and laboratory animals differ greatly in their bacterial communities, we show that the community proportional membership remains stable from Phylum to Family taxonomic levels regardless of differences in environment and diet, suggesting that endogenous factors, such as host genetics, have greater control in shaping gut community membership.

## 1 INTRODUCTION

Metazoans live symbiotically with microorganisms on and within them (Hacquard et al., 2015), and the gastrointestinal tract is one of the most studied organs for these symbiotic interactions (Douglas, 2015; Engel & Moran, 2013; Leslie & Young, 2015). Gut microbes are known to be vital for species feeding on specialized or suboptimal diets by providing essential nutrition (amino acids, vitamins) (Douglas, 2006, 2009; Wigglesworth, 1936), or aiding in degradation of otherwise indigestible plant cell walls (Douglas, 2009; Genta, Dillon, Terra, & Ferreira, 2006; Kohler, Dietrich, Scheffrahn, & Brune, 2012; Vargas‐Asensio et al., 2014). The ability of gut microbes to supplement the host genome with functional genes is believed to promote the exploitation of food previously unavailable to the host, leading to ecological isolation and divergence from those species that lack microbial symbionts (Brucker & Bordenstein, 2012; Janson, Stireman, Singer, & Abbot, 2008).

**Figure 1:**
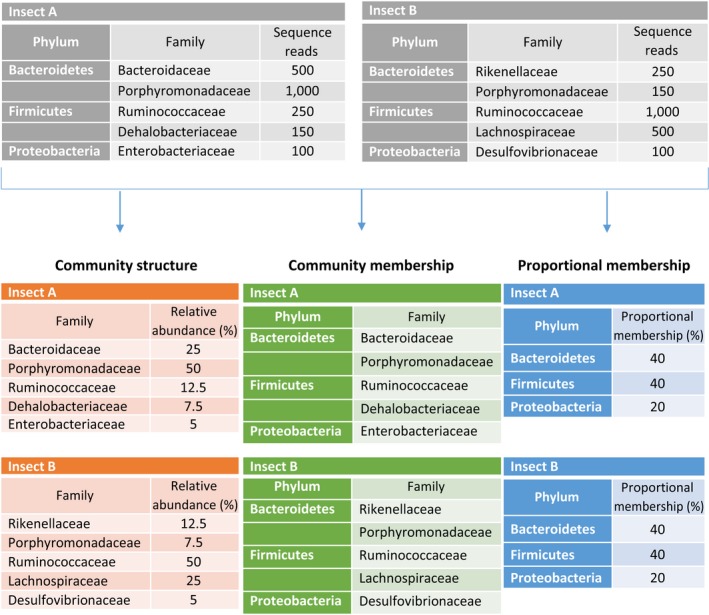
A hypothetical example of different community types in a sequencing data analysis. Sequencing results from the guts of two insects are analyzed based on the relative abundance of the sequence reads of individual taxon (Community structure), the presence or absence of microbial taxa in a sample (Community membership), and the fraction of the types of taxa that constitutes the community membership (Proportional membership). In this simplified example, Insect A and Insect B have different community structures and community memberships, as they have different abundances and different members, respectively, in the Family taxonomic level. However, both insects have identical proportional membership at Phylum level, with the same number of Family taxa under a Phylum taxon

Shifts in gut microbial communities occur in two major ways: change in community membership (presence or absence of microbial taxa) and change in community structure (*relative abundance* of microbial taxa); two communities can have the same memberships but different structures, but if memberships of communities are different, they will have different structures (Schloss & Handelsman, 2006). A related idea is the proportional membership, which is derived from a study by Zhao, Irwin, and Dong (2016); the authors counted the taxa detected and summarized the types of taxa that constitute the gut community membership as proportions at phylum level. For example, in a gut where there are 100 different bacterial species, a proportional membership of 50% for *Firmicutes* implies that 50 species are identified to that phylum. It was also demonstrated that proportional membership was consistent among different individuals in a population and showed less fluctuation than the community structure within an individual in a longitudinal survey (Zhao et al., 2016). Figure 1 uses a hypothetical example to illustrate the different descriptors of microbial communities based on sequencing data analysis and used throughout this study.

Pronounced interpopulation and interindividual variations in the gut microbial communities are observed in many species, with contributions from endogenous factors, such as age, sex and genotype, and exogenous factors, including habitat and diet (Bennett et al., 2016; Han, Lee, Jeong, Jeon, & Hyun, 2017; Kovacs et al., 2011). As gut microbes can help in the digestion of ingested food, changes in gut microbial populations could entail a shift in the genes that carry out metabolic reactions in the gastrointestinal tract, which could impact the food utilization efficiency of the host (Holm et al., 2016; Turnbaugh et al., 2006).

Host genetics are believed to influence the gut microbial community, as gut microbiota has been shown to be more similar among family members and even within populations (Turnbaugh et al., 2009; Zhao et al., 2016). But early twin studies have produced inconsistent results. For example, Stewart, Chadwick, and Murray (2005) observed a higher degree of similarity in the gut microbiota of monozygotic twins compared with dizygotic twins and unrelated pairs. Yet, it has been reported that the gut microbiota of monozygotic twins is no more similar than the microbiota of dizygotic twins (Turnbaugh et al., 2009; Yatsunenko et al., 2012). Nevertheless, recent studies, through reanalysis of previous data (Goodrich et al., 2014; Zhao et al., 2016) and genomewide association studies (Davenport, 2016; Davenport et al., 2015), provide compelling evidence that host genetics is a factor that shapes the gut microbiota. Moreover, Zhao et al. (2016) demonstrated that host genetics are fundamentally responsible for gut community membership, leaving nongenetic factors to regulate the abundance of different microbes.

Host diet is a major exogenous factor in shifting the structure of the gut bacterial community and its metabolic capabilities (Bolnick, Snowberg, Hirsch, Lauber, Knight, et al., 2014; Chandler, Lang, Bhatnagar, Eisen, & Kopp, 2011; Muegge et al., 2011). Macronutrients (proteins, carbohydrates, and lipids) in various proportions in the diet can significantly alter the gut microbiome (Daniel et al., 2014; David et al., 2014; McAllan et al., 2014; Pérez‐Cobas et al., 2015). For example, diets rich in protein reproducibly decreased the levels of *Firmicutes* that degrade plant polysaccharides in the human gut (David et al., 2014), and in mice (Kim, Kim, & Park, 2016). Similarly, high‐fat diets caused the proportion of *Ruminococcaceae* (phylum *Firmicutes*) to decrease and the proportion of *Rikenellaceae* (phylum *Bacteroidetes*) to increase (Daniel et al., 2014). Moreover, the diversity of the microbial population in the habitat could influence the types of microbes that colonize the gut. For instance, animals housed in laboratory conditions have a less diverse gut microbial community and a reduced subset of that found in their wild counterparts (Belda et al., 2011; Chandler et al., 2011; Lehman, Lundgren, & Petzke, 2009; Pérez‐Cobas et al., 2015; Staubach, Baines, Kunzel, Bik, & Petrov, 2013; Xiang et al., 2006). Yet, despite much variability in gut microbial profiles, there appears to be a core microbiome in many species (Berg et al., 2016; Pérez‐Cobas et al., 2015; Roeselers et al., 2011; Shade & Handelsman, 2012; Tinker & Ottesen, 2016; Wang et al., 2016), that is hypothesized to be the result of co‐evolution of beneficial gut microbes with their hosts (Shapira, 2016).

The effect of diet on life history traits has been well documented, especially in the trade‐offs between trait expressions due to differential allocation of limiting internal nutrients (Boggs & Ross, 1993; Cotter, Simpson, Raubenheimer, & Wilson, 2011; Kupferberg, Marks, & Power, 1994; Zera & Harshman, 2001). In recent years, crickets are emerging as a useful model organism for studying sexually selected traits and elucidating the effects of diet quality and composition on trade‐offs between life history traits and sexual traits (Gray & Eckhardt, 2001; Harrison, Raubenheimer, Simpson, Godin, & Bertram, 2014; Kelly, Neyer, & Gress, 2014; Lyn, Naikkhwah, Aksenov, & Rollo, 2011; Maklakov et al., 2008; Simmons, 2011). But few studies have examined the impact of the gut microbiome on fitness in crickets. The first report on gut microbiota of crickets dates back to 1981 (Ulrich, Buthala, & Klug, 1981), and subsequent studies between 1989 and 1998 have revealed broad categories of bacterial communities in the gut, and general changes in its composition in response to changes in diet (Kaufman & Klug, 1991; Kaufman, Klug, & Merritt, 1989; Santo Domingo, 1998; Santo Domingo, Kaufman, Klug, & Tiedje, 1998; Santo Domingo, Kaufman, Klug, Holben, et al., 1998). In addition, Kaufman and Klug (1991) found that the presence of gut bacteria increased the digestive efficiency of plant polysaccharides and allowed crickets to utilize a wider range of dietary carbohydrates. Only very recently, however, with the prevalence of next‐generation sequencing, have detailed examinations of gut microbiota been possible (Smith, Srygley, Dietrich, & Mueller, 2016; Smith, Srygley, Healy, Swaminath, & Mueller, 2017). In Mormon crickets and decorated crickets, mating, but not protein consumption, was found to influence gut microbial structure (Smith et al., 2016).

To understand how exogenous and endogenous factors influence gut bacterial communities in field crickets, we compared the gut bacterial profiles of *Teleogryllus oceanicus* exposed to different environments and diets. As expected, wild crickets had a more diverse gut microbiota than laboratory‐reared crickets, but switching the diet from standard cat chow to chemically defined diets also caused a decrease in the diversity of microbial populations in captivity. Although the abundance of various bacterial taxa was altered as a result of shifts in exogenous factors, *T. oceanicus* maintained a stable proportional membership in the gut bacterial community. Additionally, we detected changes in predicted metabolic functions between wild and laboratory‐reared crickets, due to differences in gut community structures.

## 2 METHODS

### 2.1 Cricket samples and husbandry

A total of 26 crickets from three groups were prepared: five females and two males were sampled from a natural population (wild, *n* = 7); three females and three males were sampled from laboratory stocks raised on cat chow (CC, *n* = 6); and seven females and six males were first fed cat chow and then switched to chemically defined diets at the 8th nymphal stage when sex can first be determined (CD, *n* = 13). Wild *T. oceanicus* were collected from Carnarvon in North‐Western Australia in November 2015. Laboratory crickets originated from the same location and were kept as a large outbred population (>1000 individuals), which was supplemented with wild caught crickets annually. Laboratory stocks were fed dry cat chow (Purina Friskies; 30% crude protein, 10% crude fats, 41.4% carbohydrates of grain sources) ad libitum and maintained at 26°C under a 12‐h light : 12‐h dark cycle. Laboratory crickets used in this experiment were isolated at penultimate instar in plastic containers (7 × 7 × 5 cm) and allowed ad libitum access to either cat chow (CC crickets) or chemically defined diets (CD crickets).

Two types of chemically defined diets (protein‐rich and carbohydrate‐rich) were prepared based on established protocols (Maklakov et al., 2008; Simpson & Abisgold, 1985). Briefly, they contain either protein or carbohydrate as the source of macronutrients, supplemented with fixed amounts of salts, cholesterol, and vitamins and diluted with non‐nutritional cellulose to attain a macronutrient concentration of 42% (Table 1). Crickets given chemically defined diets were able to choose freely between the two diets to regulate their protein and carbohydrate intake. On average, these crickets were observed to consume 85 ± 17 mg of protein and 247 ± 55 mg of carbohydrate over a period of 21 days.

**Table 1:** Composition of chemically defined diets

Diet components	Chemically defined diets	
	Protein‐rich	Carbohydrate‐rich
Cellulose	54	54
Casein	25.2	–
Peptone	8.4	–
Albumen	8.4	–
Sucrose	–	21
Dextrin	–	21
Linoleic acid	0.55	0.55
Cholesterol	0.55	0.55
USP XIV Salt mixture	2.5	2.5
Ascorbic acid	0.275	0.275
Vitamin mix	0.18	0.18

Diet compositions are given in percentages (g/100 g). Diets components were obtained from: Cellulose: MP Biomedicals, Cat no.: 900453; Casein: MP Biomedicals, Cat no.: 904520; Peptone: Oxoid Ltd, Cat no.: LP0037; Albumen: E.P.S. S.P.A.; Sucrose: MP Biomedicals, Cat no.: 904713; Dextrin: MP Biomedicals, Cat no.: 960376; Linoleic acid: Sigma‐Aldrich, Cat no.: 62240; Cholesterol: MP Biomedicals, Cat no.: 101380; USP XIV Salt mixture: MP Biomedicals, Cat no.: 902850; Ascorbic acid: Chem‐Supply Pty Ltd, Cat no.: AL022; Vitamin mix: Sigma‐Aldrich, Cat no.: V1007.

At day 21 of adult age, laboratory‐reared crickets were kept without food for 15–17 hr, to clear the gut of residual food and nonresident microbes, before they were freeze‐killed and stored at −20°C until DNA extraction. Similarly, crickets caught from the wild were kept without food before they were freeze‐killed. Although the age of our wild crickets was not known, previous field studies have shown that wild field crickets are unlikely to be older than 21 days (Murray & Cade, 1995; Simmons & Zuk, 1994; Zuk, 1987).

### 2.2 DNA extraction and sequencing of bacterial 16S ribosomal RNA genes

Crickets were immersed in 70% ethanol for 5 min and rinsing with sterile water before the midgut and hindgut were dissected and homogenized aseptically. DNA was extracted using a PowerSoil DNA Isolation Kit (MO BIO Laboratories, Inc., Carlsbad, CA) according to the manufacturer's protocol with the following modifications (Bolnick, Snowberg, Hirsch, Lauber, Org, et al., 2014). Samples were incubated at 65°C for 10 min after Solution C1 was added and vortexed horizontally at maximum speed for 2 min. The final elution step was carried out twice with 50 μl of Solution C6, with a 5‐min incubation for each elution.

Amplification of bacterial 16S rRNA was performed in a single round of polymerase chain reaction (PCR) using fusion tag primers consisting of Illumina adaptors, indexes unique to this study, and the template‐specific primers 515F (5′‐ GTGCCAGCMGCCGCGGTAA ‐′3) and R806 (5′‐ GGACTACHVGGGTWTCTAAT ‐′3) in an ultra‐clean laboratory at Curtin University (Caporaso et al., 2011; Turner, Pryer, Miao, & Palmer, 1999). 16S rRNA amplicons were generated in a single amplification step to minimize the impacts of chimeras and contamination. PCR reagents included 1 × AmpliTaq Gold® Buffer (Life Technologies), 2 mM MgCl_2_, 0.25 μM dNTPs, 10 μg BSA, 5 pmol of each primer, 0.12 × SYBR® Green (Life Technologies), 1 Unit AmpliTaq Gold DNA polymerase (Life Technologies), 2 μl of DNA, and Ultrapure™Distilled Water (Life Technologies) made up to 25 μl. PCR was executed on an Applied Biosystems StepOnePlus Real‐Time PCR system under the following conditions: initial denaturation at 95°C for 5 min, followed by 35 cycles of 30 s at 95°C, 30 s at 50°C, and 45 s at 72°C and completed with a final extension for 10 min at 72°C. Duplicates originating from each sample were combined prior to amplicon pooling and library preparation.

The amplicon library for sequencing was prepared by pooling PCR products into equimolar ratios based on qPCR and quantification using a Labchip® GX Touch HT (Perkin Elmer). To assess cross‐contamination, PCR and DNA extraction controls were also included in the final library for sequencing. Amplicons in the library were size‐selected using a Pippin Prep (Sage Science) and purified using the Qiaquick PCR Purification Kit (Qiagen). The volume of purified library added for sequencing was determined using a Labchip® GX Touch HT (Perkin Elmer) and sequenced (uni‐directionally) using a 300 cycle MiSeq® v2 Reagent Kit and standard flow cell on an Illumina MiSeq platform located in the TrEnD Laboratory at Curtin University.

### 2.3 Data analysis

To ensure high‐quality sequences were generated, which translate into robust OTU‐based analyses, a series of quality control steps were undertaken. This included (1) using primer/index combinations that have never been previously used in the laboratory to reduce the risk of contamination, (2) generating the sequencing library in a single round of PCR to minimize the risk of contamination and reduce the likelihood of chimeric sequences, (3) only including sequences that, using Geneious V8.1.4 (Kearse et al., 2012), have a 100% identity match to the Illumina adaptor, index barcodes and the template specific primer sequences, (4) sequencing negative controls and removing those reads across samples accordingly, (5) denoising sequences which further collapses similar sequences together (that could arise from PCR or sequencing error), (6) intrasample chimera checking, and (7) removing singletons that could arise from PCR or sequencing error and those with any ambiguous base calls.

Sequence data were processed with the mothur software package version 1.38.0 (Schloss et al., 2009), according to the MiSeq standard operating procedure, with some modifications (Kozich, Westcott, Baxter, Highlander, & Schloss, 2013; Schloss & Westcott, 2016). The mothur commands used in the analysis are provided in detail in the Supplementary Methods. Briefly, sequences with any ambiguous bases or sequence lengths that were either shorter than 240 bp or longer than 260 bp were removed; processed sequences were collapsed to unique sequences, which were aligned to the SILVA reference database (Release 123) (Pruesse et al., 2007; Quast et al., 2013). Aligned sequences were screened for chimeras using UCHIME (Edgar, Haas, Clemente, Quince, & Knight, 2011) (quality control step 6); taxonomic classification of sequences was based on the Greengenes reference database (May 2013 release) (DeSantis et al., 2006; McDonald et al., 2012) using the Wang method. Unidentifiable sequences or sequences classified as *Eukaryota*, chloroplasts, mitochondria, or *Wolbachia* (endosymbiont) were removed. Low abundance sequences (singletons, doubletons, and tripletons) could potentially reflect the rare biosphere of a microbial community (D Ainsworth et al., 2015), but to avoid inflating the actual microbial diversity in the cricket gut due to PCR or sequencing errors (Dickie, 2010; Kunin, Engelbrektson, Ochman, & Hugenholtz, 2010), singletons were excluded from downstream analyses (Beckers, Op De Beeck, Weyens, Boerjan, & Vangronsveld, 2017; Tedersoo et al., 2010) (quality control step 7). Finally, a total of nineteen OTUs, with a maximum of three sequence reads for any individual OTU, were obtained from all the negative controls. Consequently, sequences in cricket samples were only considered as contaminants when they were classified to the OTUs found in the negative controls, and when their abundances were less than 30 reads per sample in all crickets.

Rarefaction curves and alpha diversity indices (inverse Simpson's index and Chao1 index) were calculated using mothur commands (Schloss & Westcott, 2016). Bray–Curtis distance metric, unweighted and weighted Unifrac distance metrics were used to estimate the beta diversity and visualized with PCoA. Bray–Curtis distance metric was performed on relative sequence abundances after square root transformation. Commands in mothur (clearcut, unifrac.unweighted, and unifrac.weighted) were used for Unifrac distance metrics. PERMANOVA using the vegan package in R was used to determine the significance of clustering in the PCoA plots, and betadisper() function was used to determine the homogeneity of dispersion among cricket groups (*F*(2,23) = 1.5256, *p* = .235). To identify the OTUs that characterize the differences among the three groups, LEfSe (Segata et al., 2011) was performed using the lefse command and default parameters in mothur (Schloss & Westcott, 2016). Bacterial community proportional membership summary was calculated as the counts of taxa at each taxon level and expressed as proportions in each sample (Zhao et al., 2016). Compositional or proportional data were analyzed using Aitchison geometry (Aitchison, 1986) of compositions package (van den Boogaart & Tolosana‐Delgado, 2008) in R. Graphs were constructed using Microsoft Excel and R.

### 2.4 Predictive metabolic capabilities of cricket gut microbiota

After closed‐reference OTU picking was used to exclude unclassified OTUs, 99.6% of the sequences were retained for predictive functional profiling. PICRUSt v1.1.0 (Langille et al., 2013) was used to predict gene families of the bacterial communities in cricket guts, according to the online protocol (http://picrust.github.io/picrust/index.html), by referencing the sequenced 16S rRNA gene data to KEGG Orthology Database (Kanehisa & Goto, 2000). PICRUSt output was analyzed and visualized with STAMP version 2.1.3 (Parks, Tyson, Hugenholtz, & Beiko, 2014). Statistical significance was calculated using ANOVA and Tukey–Kramer method for post hoc tests, with confidence intervals set to 0.95; Benjamini–Hochberg false discovery rate (FDR) was used for multiple test correction.

## 3 RESULTS

### 3.1 Characterization of *T. oceanicus* gut microbiota

**Figure 2:**
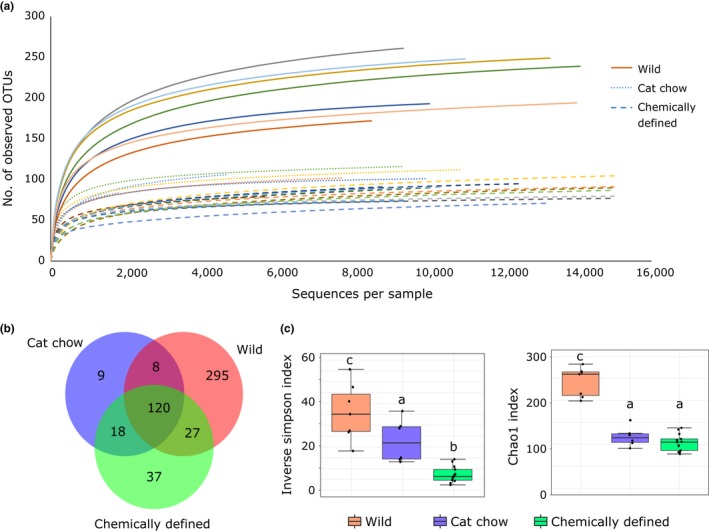
Diversity of gut microbiota in crickets. (a) Rarefaction curves of 16S rRNA genes from 26 crickets. (b) Venn diagram depicting the distribution of the total 514 operational taxonomic units (OTUs) identified. Wild crickets (*n* = 7) have more unique OTUs than laboratory‐reared crickets fed cat chow (*n* = 6) or chemically defined diets (*n* = 13). (c) Alpha diversity indices, inverse Simpson's index (left panel), and Chao1 index indicate that wild crickets have greater diversity in their gut bacterial communities. Boxes cover the interquartile range (IQR) and the line inside the box denotes the median. Whiskers represent the lowest and highest values within 1.5 × IQR. Analysis of variance (ANOVA): inverse Simpson's index—*F*(2,23) = 26.52, *p* < .0001; Chao1 index—*F*(2,23) = 77.21, *p* < .0001. Different small letters signify significant differences in Tukey's honest significant difference (HSD) post hoc tests

After quality filtering and removal of unintended sequences, 319,616 16S rRNA sequences were obtained from 26 samples, with a mean (± standard deviation) of 12293 ± 5752 reads per sample that resulted in 514 operational taxonomic units (OTUs) at 97% nucleotide similarity. Rarefaction curves of all samples reached saturation plateaus, indicating that the sequencing depth was sufficient to capture most bacterial species in the gut (Figure 2a). Of the 514 OTUs, 450 OTUs were found in wild crickets, 155 OTUs in laboratory‐reared crickets fed cat chow (CC), and 202 OTUs in laboratory crickets fed chemically defined diets (CD) (Figure 2b). Besides having at least twice as many OTUs, wild crickets also had more unique OTUs than CC and CD crickets (Figure 2b). However, those 295 unique OTUs comprised only 29% of the total reads in wild crickets; in contrast, 57.2% of their sequences were assigned to the 120 OTUs that were shared among the three cricket groups. This suggested that *T. oceanius* shared a large portion of their gut microbiota, despite being raised in different environment and fed different diets. Alpha diversity indices also implied that the gut microbiota of wild crickets was more diverse (inverse Simpson's index: *F*(2,23) = 26.52, *p* < .0001; Chao1 index: *F*(2,23) = 77.21, *p* < .0001; Figure 2c). Laboratory‐reared crickets had similar species richness (Chao1 index; Figure 2c), but CC crickets had greater species evenness than CD crickets (inverse Simpson's index; Figure 2c).

**Figure 3:**
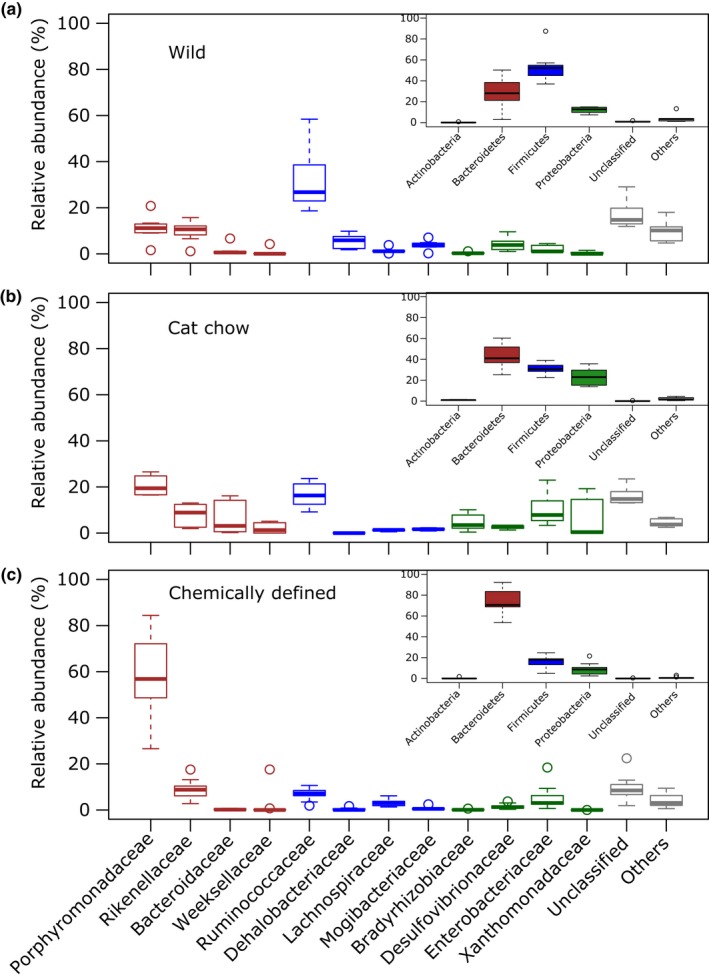
Gut bacterial community structure (relative abundance in microbial taxa). (a–c) Relative abundances of gut bacteria by Phylum (inserts) and Family level in crickets (a) captured from field (*n* = 7), (b) given cat chow (*n* = 6) and, (c) given chemically defined diets (*n* = 13). Boxplots of bacterial Families from respective Phyla are outlined in the same color; boxes cover the interquartile range (IQR) and the line inside the box denotes the median. Whiskers represent the lowest and highest values within 1.5 × IQR. Detailed taxonomic classification and abundances are listed in Table S1

Taxonomic classification yielded 12 different bacterial phyla (Table S1 ), but the predominant phyla in *T. oceanicus* of all three groups were *Bacteroidetes*,* Firmicutes,* and *Proteobacteria* (Figure 3a–c inserts), accounting for 97.3% ± 3% of the total sequences in each cricket. On average, wild crickets had a higher percentage of *Firmicutes* (54% ± 16%) in the gut than laboratory‐reared crickets (CC: 31% ± 5%; CD: 16% ± 5%). In CD crickets, *Bacteroidetes* represented 74% ± 10% of the total bacteria in the gut, nearly twice as much compared to wild (29% ± 16%) and CC (42% ± 12%) crickets. *Bacteroidetes* to *Firmicutes* ratios were significantly different between CD and wild crickets (*t*(23) = −2.439, *p* = .0228), but not between CD and CC crickets (*t*(23) = −0.283, *p* = .7796). Besides having abundance of *Bacteroidetes* and *Firmicutes* intermediate of wild and CD crickets, CC crickets had more *Proteobacteria* than the other cricket types. In addition, wild crickets had five bacterial phyla (*Cyanobacteria*,* Fusobacteria*,* Lentisphaerae*,* Planctomycetes*, and *Synergistetes*) that were not detected in laboratory‐reared crickets (Table S1).

At family level, *Porphyromonadaceae* was highly represented in CD crickets, accounting for 60% of the total gut bacteria (Figure 3c). However, *Ruminococcaceae* from the phylum *Firmicutes* was the prevalent family taxon in wild crickets (Figure 3a). In contrast to wild and CD crickets, CC crickets had similar percentages of *Porphyromonadaceae* and *Ruminococcaceae*, and an increased abundance in *Bacteroidaceae* from the phylum *Bacteroidetes*, and *Bradyrhizobiaceae*,* Enterobacteriaceae* and *Xanthomonadaceae* from the phylum *Proteobacteria* (Figure 3b).

**Figure 4:**
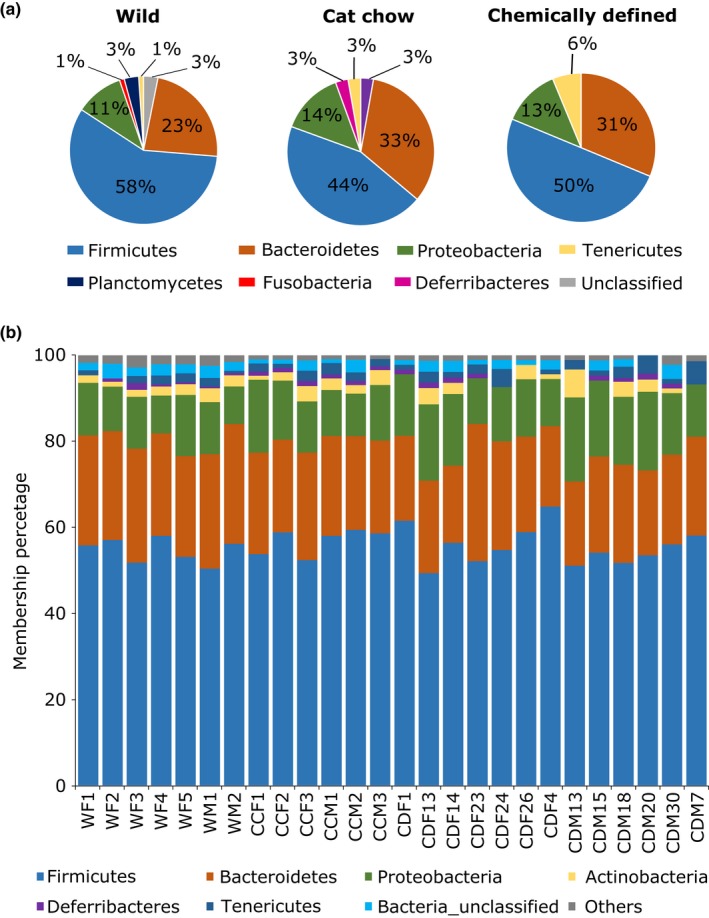
Gut bacterial community proportional membership. (a) Community proportional membership of linear discriminant analysis effect size (LEfSe) identified OTUs assigned to Phyla level. (b) Community proportional membership of bacterial Phyla across all samples. Taxa with less than 1% membership in samples of each cricket group are grouped within Others. No significant difference was detected among diet groups (PERMANOVA;* p* = 0.216, *R* ^2^ = .108). WF, wild female cricket; WM, wild male cricket; CCF, cat chow female cricket; CCM, cat chow male cricket; CDF, chemically defined diet female; cricket CDM, chemically defined diet male cricket

At the OTU level, wild crickets had an average of 222 ± 35 OTUs, CC crickets had 103 ± 11 OTUs, and CD crickets had 88 ± 11 OTUs. Linear discriminant analysis effect size (LEfSe) linked 95 OTUs, 36 OTUs, and 16 OTUs that were significantly enriched to wild crickets, CC crickets, and CD crickets, respectively (LDA > 2, *p* < .05; Table S2). In agreement with diversity analysis (Figure 2), CD crickets had fewer characterizing OTUs, and the number of phyla corresponding to LEfSe‐identified OTUs was lower than wild and CC crickets (CD – 4 phyla, Wild – 7 phyla, CC – 6 phyla; Figure 4a). However, in contrast to relative abundance of gut bacterial composition (Figure 3a–c), LEfSe identified similar proportions of major phyla membership (presence or absence of taxa) in all cricket groups; *Firmicutes*,* Bacteroidetes*, and *Proteobacteria* represented around 50%, 30%, and 13% of the LEfSe‐identified OTUs, respectively (Figure 4a). This proportional membership was also observed in the full 16S rRNA data from Phylum to Family taxonomic levels (Figure 4b; Fig. S1–3).

**Figure 5:**
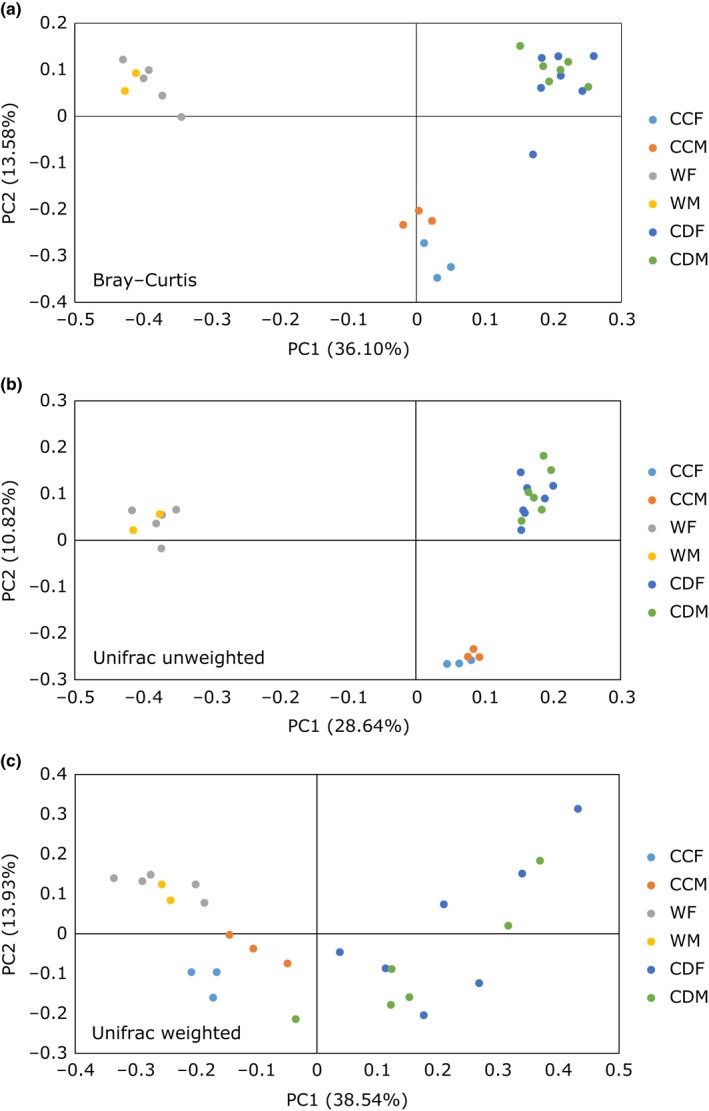
Principal coordinates analysis (PCoA) of 26 cricket samples. Crickets in different groups clustered independently based on (a) Bray–Curtis distance matrices after square root transformation of relative sequence abundance data (PERMANOVA;* p* < 0.001, *R* ^2^ = .48), (b) unweighted Unifrac distance metrics (*p* < .001, *R* ^2^ = .39), and (c) weighted Unifrac distance metrics (*p* < .001, *R* ^2^ = .44). CCF, cat chow female cricket; CCM, cat chow male cricket; WF, wild female cricket; WM, wild male cricket; CDF, chemically defined diet female cricket; CDM, chemically defined diet male cricket

Principal coordinates analysis (PCoA) using Bray–Curtis distance metric (BC), unweighted (uwU) and weighted (wU) Unifrac distance metrics illustrated that crickets of the three different groups clustered independently (Figure 5). Permutational multivariate analysis of variance (PERMANOVA) showed significant differences among clusters of cricket types for all distance metrics (BC: *p* < .001, *R* ^2^ = .48; uwU: *p* < .001, *R* ^2^ = .39; wU: *p* < .001, *R* ^2^ = .44), demonstrating that the community membership and structure were different for the three cricket groups. There was no significant effect of sex on gut microbiota (BC: *p* = .095, *R* ^2^ = .035; uwU: *p* = .288, *R* ^2^ = .028; wU: *p* = .583, *R* ^2^ = .018). However, sample sizes for sex differences were very small and would need to be increased to confirm the lack of sexual difference in the gut microbiota of these crickets.

### 3.2 Core gut microbiota of *T. oceanicus*

To determine the presence of a core microbiota that is resistant to changes in diet and environment, OTUs shared by all samples were considered (100% core threshold) (Otani et al., 2014). Of the 514 OTUs identified, 10 OTUs from the phyla *Bacteroidetes* and *Firmicutes*, and distributed among 5 families, were found in all crickets examined (Table 2). They contributed to around 20% of the total sequences found in wild and CC crickets and to nearly 50% in CD crickets. Multivariate analysis of variance (MANOVA) showed a significant effect of diet on the difference of core taxa (*F*(2,23) = 1.3824, *p* = .00033).

**Table 2:** Relative abundance of core operational taxonomic units (OTUs) (100% threshold) in wild, cat chow (CC) and chemically defined diet (CD) crickets. Values are mean relative abundance with standard deviation

OTU	Phylum	Family	Relative abundance (%)		
			Wild crickets	CC crickets	CD crickets
Otu0001	Bacteroidetes	Porphyromonadaceae	0.75 ± 0.99	2.04 ± 1.47	20.54 ± 19.5
Otu0002	Bacteroidetes	Porphyromonadaceae	0.69 ± 0.57	2.63 ± 1.9	19.32 ± 14.04
Otu0006	Firmicutes	Ruminococcaceae	6.49 ± 4.42	4.85 ± 4.11	0.85 ± 0.56
Otu0007	Bacteroidetes	Rikenellaceae	0.14 ± 0.17	0.87 ± 0.64	4.46 ± 3.41
Otu0008	Firmicutes	Ruminococcaceae	5.32 ± 2.89	1.4 ± 0.52	1.51 ± 0.88
Otu0011	Firmicutes	Ruminococcaceae	4.06 ± 3.03	1.26 ± 0.65	0.83 ± 0.9
Otu0014	Bacteroidetes	Rikenellaceae	1.24 ± 1.56	2.11 ± 1.79	1.07 ± 1.55
Otu0015	Firmicutes	Mogibacteriaceae	2.7 ± 1.98	0.99 ± 0.49	0.43 ± 0.51
Otu0036	Bacteroidetes	Rikenellaceae	0.35 ± 0.47	0.97 ± 0.67	0.58 ± 0.4
Otu0047	Firmicutes	Ruminococcaceae	0.96 ± 0.39	0.51 ± 0.49	0.31 ± 0.24
Average proportion of total reads			22.71	17.63	49.89

### 3.3 Predictive metabolic capabilities

Gut microbes could provide the host with additional enzymatic genes and aid in digestion of ingested food (Douglas, 2009; Genta et al., 2006; Kohler et al., 2012; Vargas‐Asensio et al., 2014). To determine if alteration of the gut bacterial composition under different conditions could translate to differences in metabolic capabilities, 16S rRNA gene sequence data were subjected to Phylogenetic Investigation of Communities by Reconstruction of Unobserved States (PICRUSt) analysis to predict the metabolic profile of the gut bacterial communities. The average weighted nearest sequenced taxon index (NSTI) for all samples was 0.12 ± 0.01, similar to a previous study of mammals and in range of useful predictions despite fewer available reference genomes (Langille et al., 2013).

**Figure 6:**
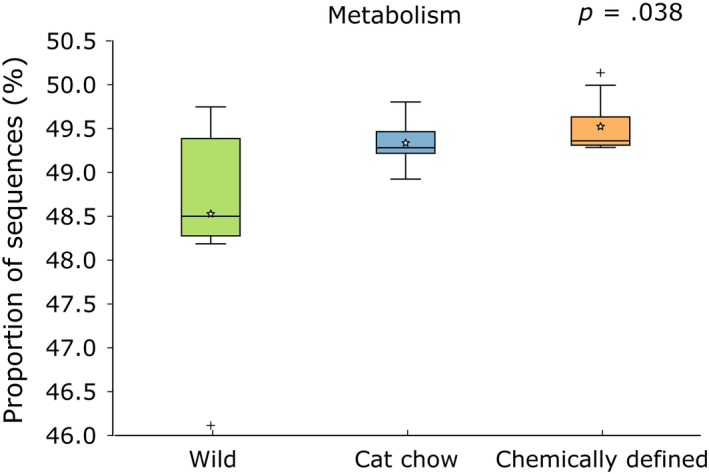
Predicted metagenomes of gut bacterial communities in crickets. Proportion of 16S rRNA gene sequences assigned to the Metabolism category at Kyoto Encyclopedia of Genes and Genomes (KEGG) Level 1 (Mean: Wild (*n* = 7) – 48.5%, Cat chow (*n* = 6) – 49.3%, Chemically defined (*n* = 13) – 49.5%). Boxes cover the interquartile range (IQR) and the line inside box denotes the median. Star represents the mean of data, and whiskers represent the lowest and highest values within 1.5 × IQR

**Figure 7:**
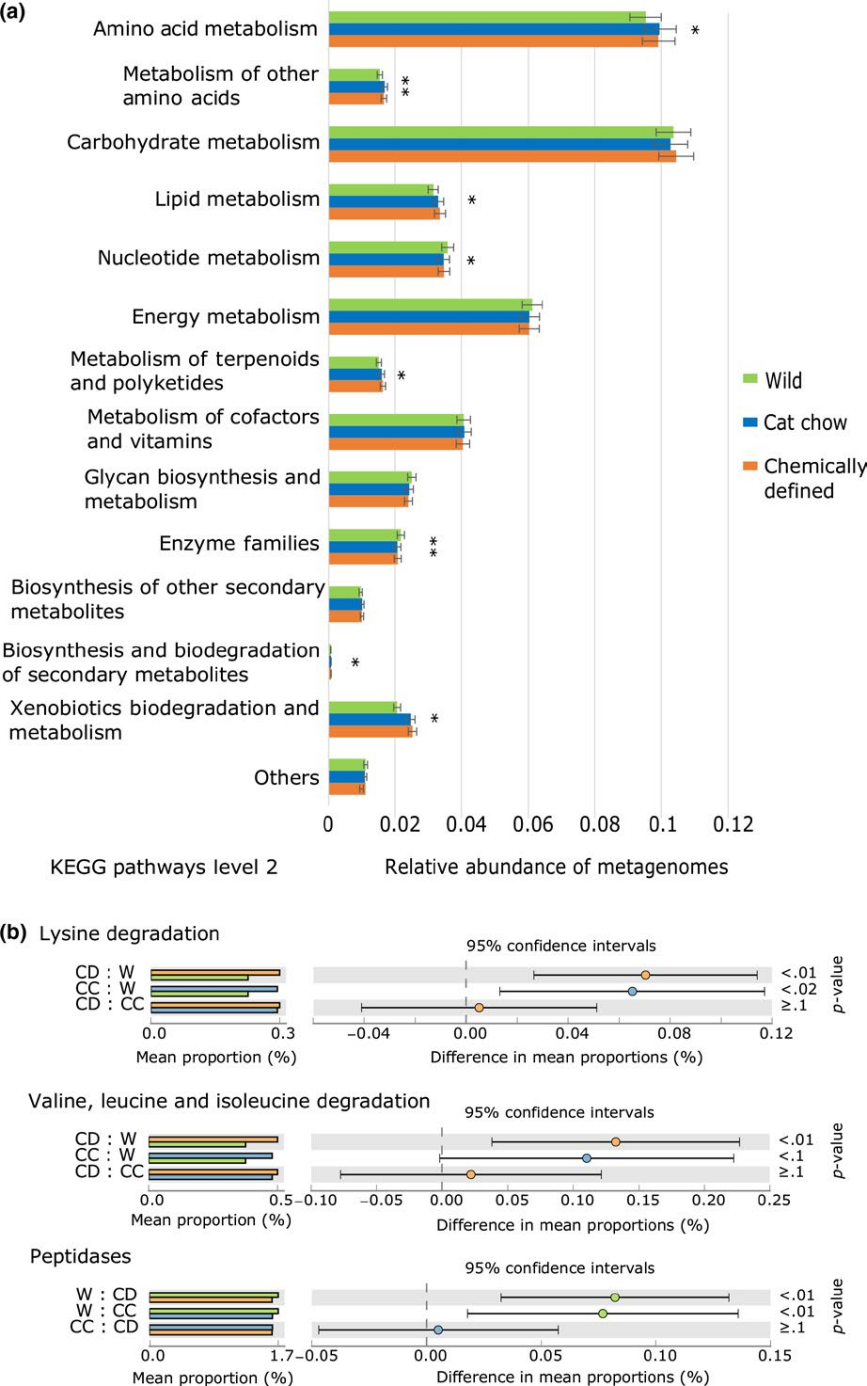
Predicted Kyoto Encyclopedia of Genes and Genomes (KEGG) Level 2 and Level 3 metagenomes. (a) Eight of fourteen categories in KEGG Level 2 of Metabolism are significantly different among the three cricket groups. *p < .05, **p < .001. (b) Chemically defined diet (CD) and cat chow (CC) crickets have more sequences assigned to degradation of essential amino acids. Wild (W) crickets, on the other hand, possess more peptidases within KEGG Level 3 categories. Refer to Table S3 for full list of KEGG Level 3 categories. Analysis of variance (ANOVA) and Tukey–Kramer post hoc tests are performed with STAMP v2.1.3, along with Benjamini–Hochberg false discovery rate (FDR) for multiple est corrections

There was a small but significant difference in the proportion of sequences assigned to Metabolism in Kyoto Encyclopedia of Genes and Genomes (KEGG) Level 1 categories (ANOVA, *p* = .038; Figure 6); Tukey–Kramer post hoc tests revealed that CD crickets were significantly different from wild profiles (*p* < .02). In KEGG Level 2 categories of Metabolism, eight KEGG pathways were significantly different among the three cricket groups, including amino acid metabolism and lipid metabolism (Figure 7a); post hoc tests indicated that wild crickets were significantly different from both CD crickets and CC crickets in the eight KEGG Level 2 categories, but not between CD and CC crickets (Fig. S4). In KEGG Level 3 subcategories, CD and CC crickets had higher proportion of sequences assigned to degradation of essential amino acids, while wild crickets had higher abundance of peptidases (Figure 7b). CD and CC crickets also had significantly higher proportion of sequences assigned to the metabolism of other amino acids (glycine, serine, threonine, phenylalanine, tryptophan, and tyrosine) and to the biosynthesis of unsaturated fatty acids and lipids (Table S3).

## 4 DISCUSSION

The metazoan gut is colonized by an opportunistic and commensal microbiota that is shaped by a combination of exogenous (diet and habitat) and endogenous (gut environment and host genetics) factors (Bennett et al., 2016; Bolnick, Snowberg, Hirsch, Lauber, Knight, et al., 2014; Bolnick, Snowberg, Hirsch, Lauber, Org, et al., 2014; Daniel et al., 2014; David et al., 2014; Dehler, Secombes, & Martin, 2017; Muegge et al., 2011; Pérez‐Cobas et al., 2015; Yun et al., 2014). It has been suggested that different factors act on different aspects of gut microbial community composition (Zhao et al., 2016). Here, we described the gut microbiota of *T. oceanicus* and investigate how changes in environment and diet affect gut bacterial profiles.

Our results demonstrate that the gut microbiota of wild crickets was more diverse than their laboratory‐reared counterparts, confirming observations in a range of arthropod species (Belda et al., 2011; Pérez‐Cobas et al., 2015; Staubach et al., 2013; Xiang et al., 2006). Wild *T. oceanicus* had more unique OTUs and five bacterial phyla (of 12 phyla identified in all samples) not detected in the laboratory‐reared crickets. As our laboratory cricket population is supplemented with wild crickets annually, it would seem that the yearly reintroduction of additional gut microbial species does not persist in the laboratory environment. While the decrease in gut bacterial diversity in laboratory crickets could be due to reduced constant exposure to diverse environmental microbes (relatively cleaner laboratory environment and food), CD crickets had a lower bacterial diversity than CC crickets. It should be noted that laboratory (CC and CD) crickets in our experiments were fed the same cat chow during their nymphal stages. CD crickets were only given chemically defined diets from penultimate instar onwards. Therefore, the less diverse gut microbiota in CD crickets could arise from (1) incomplete retention of gut microbiota as they molt to adult, (2) gradual loss of bacterial species due to lack of continual input from cat chow, (3) changes in nutritional content in the chemically defined diets that affect the survival of existing gut microbes, or a combination of these factors.

The most dominant phyla in the gut of all crickets examined (wild and laboratory‐reared) were *Bacteroidetes*,* Firmicutes,* and *Proteobacteria*, which are also prevalent in most insect orders and species examined (Colman, Toolson, & Takacs‐Vesbach, 2012; Yun et al., 2014), including orthopteran species (Smith et al., 2016, 2017; Waite et al., 2015; Yun et al., 2014) and omnivorous cockroaches (Pérez‐Cobas et al., 2015; Tinker & Ottesen, 2016), and in the guts of mammals (Ley et al., 2008). However, the three cricket groups had contrasting abundances of the dominant taxa and clustered independently in the PCoA plots. Wild crickets had higher abundance of *Firmicutes* and *Ruminococcaceae*, while those fed chemically defined diets had higher amount of *Bacteroidetes* and *Porphyromonadaceae*. Crickets fed cat chow had *Firmicutes*,* Bacteroidetes*,* Porphyromonadaceae,* and *Ruminococcaceae* abundances intermediate between wild and CD crickets, and a greater abundance of *Proteobacteria*. It has been shown that bacterial abundances are associated with macronutrient content in diets (Daniel et al., 2014; David et al., 2014; McAllan et al., 2014; Pérez‐Cobas et al., 2015). For example, animals that consume high protein/low carbohydrate or animal‐based diets have a lower abundance of polysaccharide‐degrading *Firmicutes* and a higher abundance of *Bacteroidetes* (David et al., 2014; Kim et al., 2016).

Wild field crickets are omnivorous and their natural diets are extremely varied (Gangwere, 1961; Huber, Moore, & Loher, 1989), but the higher abundance of *Firmicutes* and *Ruminococcaceae* in the gut would suggest that they consume more of a plant‐based diet. The correlation of a herbivorous diet with higher *Firmicutes* to *Bacteroidetes* ratio was also observed in another orthopteran species, the Auckland tree weta (Waite et al., 2015), which are considered to be herbivores and opportunistic omnivores (Griffin, Morgan‐Richards, & Trewick, 2011). The decrease in *Firmicutes* to *Bacteroidetes* ratio observed in CC crickets is likely due to a change in diet to cat chow, which tends to have a higher protein to carbohydrate ratio. The lower *Firmicutes* to *Bacteroidetes* ratios observed in CD crickets could be partly explained by the switch to a simpler chemically defined diet that contained only protein and carbohydrates, but no fats, as the source of macronutrients. However, another possible reason for the sheer abundance of *Bacteroidetes* in CD crickets may be the high amount of cellulose in chemically defined diets (Table 1). The two most abundant bacteria in CD crickets (making up nearly 40% of their total bacterial population) were identified to the bacterial genus *Parabacteroides* (Phylum: *Bacteroidetes*, Family: *Porphyromonadaceae*; Table 2), which has been shown to grow well in cultures containing cellulose (Gupta et al., 2014; Ziemer, 2014). Nonstarch polysaccharides, such as cellulose, pectins, and chitins, are known to act as prebiotics that help the growth of beneficial gut bacteria and induce changes in gut bacterial populations (Laparra & Sanz, 2010). Nonetheless, the exact reason for the increased abundance of *Parabacteroides* and whether these specific gut bacterial species are beneficial to the crickets require further study.

The community membership and structure were different for the three cricket groups, as determined by Bray–Curtis and Unifrac distance metrics. This is expected as the crickets fed cat chow and chemically defined diets had fewer OTUs and were characterized by a less diverse microbiota. But, interestingly, all three cricket groups possessed similar bacterial community proportional memberships in their guts, regardless of the differences in diet and environment (Figure 4b; Fig. S1–S3). LEfSe analysis uses relative abundances of gut microbiota to identify biomarkers that characterize the differences between two or more groups (Segata et al., 2011). It detected OTUs that were significantly enriched in each of the cricket groups and the phylum proportional membership of LEfSe‐identified OTU remained the same (Figure 4a). This demonstrates that, despite the fluctuation in bacterial species abundance, the gut microbiota in *T. oceanicus* preserved a stringent qualitative proportional membership. This is in agreement with the work of Zhao et al. (2016), who hypothesized that the gut community membership is controlled by host genetics, while nongenetic factors influence the abundance of each taxon. Moreover, the maintenance of such a proportional membership in a gut bacterial community has implications in microbial ecology. For instance, certain bacterial species may have “keystone functions” that alter the physical space, colonization sites, or the resources within niches in the gut to allow interacting bacterial species to flourish (Messer, Liechty, Vogel, & Chang, 2017). Different OTUs of the same phylum enriched in different cricket groups reflect their varying ability to adapt to changes in the gut environment. But, functional redundancy, where loss of a bacterial species could be replaced by another with similar functions, within members of a phylum permits functional stability of gut microbiota in a continuous state of disturbance (Lozupone, Stombaugh, Gordon, Jansson, & Knight, 2012; Mahowald et al., 2009; Moya & Ferrer, 2016). Therefore, dynamic shifts in community structure allow the host and the gut microbes to respond to constant change in the environment, yet stable proportional membership maintains the complex web of symbiotic interactions in a community. However, it is not known what keystone function they might be offering, if any. Likewise, whether the absolute abundance of keystone species, or some threshold abundance is critical to the stability and resilience of gut microbiota remains to be studied. It would also be interesting to determine if resilient community proportional membership is similarly maintained in other animals.

It has been suggested that shifts in the gut microbial composition could allow animals to adapt to changing environments and/or allow them to colonize previously inhabitable areas by helping the hosts to digest consumed food more efficiently to meet their nutrient demands (Amato et al., 2014, 2015; Brucker & Bordenstein, 2012; Douglas, 2009; Genta et al., 2006; Janson et al., 2008; Kohler et al., 2012; Vargas‐Asensio et al., 2014). A higher ratio of *Firmicutes* to *Bacteroidetes* is linked to an increased capacity for energy harvest (Turnbaugh et al., 2006). In black howler monkey, for instance, an increased abundance of *Ruminococcaceae* during seasonal shift of diet from fruits to leaves was found to enhance fermentation of plant carbohydrates to produce energy‐rich short‐chain fatty acids (Amato et al., 2015). Given that environment and diet influenced the relative abundances of microbial taxa in the gut of *T. oceanicus*, the metabolic functions of gut bacteria are likely to vary. Indeed, PICRUSt analysis predicted differences in metabolic capabilities of gut microbiota between wild and laboratory‐reared crickets. We are cautious in accepting this interpretation because of the relatively high value of NSTI, and the fact that we did not quantify gene expression or microbial products (i.e., enzymes or short‐chain fatty acids) in this study. Although *Ruminococcaceae* has been shown to play a key role in polysaccharide degradation (Flint, Scott, Duncan, Louis, & Forano, 2012), there was no significant difference in the carbohydrate metabolism between wild and CD crickets, despite huge differences in abundance. However, this could be compensated by the sheer abundance of *Porphyromonadaceae* in crickets on a CD diet, as there is evidence that *Porphyromonadaceae* also possesses genes involved in the degradation of complex carbohydrates (Hahnke et al., 2015), resulting in convergence of functions even though there was a difference in gut microbial composition (Bletz et al., 2016; Muegge et al., 2011). Based on the decrease of *Firmicutes* to *Bacteroidetes* ratio observed from wild crickets to those fed on CC and CD diets, we predict a shift in diet from one that is high in plant material in field populations to a laboratory diet higher in protein (David et al., 2014). Indeed, the cat chow used in our laboratory contains 30% protein, and CD crickets consumed, on average, 14.5% protein. Future studies using the geometric framework for nutrition would provide a good framework with which to test for changes in specific bacterial taxa in relation to varying protein consumptions (Holmes et al., 2017).

The concept of a core microbiota has been suggested to be the product of constant interactions among the diverse gut bacterial species and with their host, resulting in the symbiotic relationship of stable and resilient communities within the host (Messer et al., 2017; Shapira, 2016). There were only 10 OTUs common to all samples that we considered to be the core species in *T. oceanicus* gut microbiota, but most of the families in the core OTUs (*Porphyromonadaceae*,* Ruminococcaceae*, and *Rikenellaceae*) are also present in the core composition of omnivorous cockroaches, *Blattella germanica* (Pérez‐Cobas et al., 2015) and *Shelfordella lateralis* (Schauer, Thompson, & Brune, 2014). Convergence of gut microbial profiles due to similar diet across phylogenetically distant lineages of different orders has been observed in mammals (Bittleston, Pierce, Ellison, & Pringle, 2016; Delsuc et al., 2014; Muegge et al., 2011). Whether omnivorous feeding habits drive convergence of gut microbial profiles, and convergence of functions, across insects generally requires further study.

In conclusion, we describe the gut microbiota of three groups of field crickets feeding on different diets. We found a reduction in microbiota diversity from wild crickets to populations maintained under laboratory conditions and in laboratory‐reared crickets that had switched from standard cat chow to chemically defined diets. We confirmed exogenous factors are determinants of the abundances of various gut bacteria in *T. oceanicus*. But, this is, to our knowledge, the first study to demonstrate stable community proportional membership in both wild and laboratory‐reared crickets, despite dynamic shifts in community structure. Based on the gut microbial profile, we predict that the natural diet of wild crickets, which has always been a mystery, to be more plant‐based and low in protein. Future studies examining the gut bacterial compositions in relation to varying protein consumptions could determine the presence of gut microbial‐macronutrient signatures in crickets. We also found 10 core OTUs that were present in all crickets sampled, which are similar to those found in the omnivorous cockroaches (Pérez‐Cobas et al., 2015; Schauer et al., 2014), and comparative studies of the gut microbiota in different omnivores could reveal their contributions to the evolution of omnivorous diets. Lastly, why the diversity of gut microbiota is dramatically reduced in captive/laboratory animals has remained largely unexplained. It may be that captive animals lose gut bacteria and replace them with bacteria from their novel environment and food. It remains to be seen whether the captive animals are able to regain their novel gut microbial profile after released back to the wild. Our study system could be potentially useful as a comparative model for monitoring the gut health of animals under captive breeding and reintroduction programs.

## CONFLICT OF INTEREST

The authors declare no competing financial interests.

## DATA ACCESSIBILITY

The raw sequences reported in this study are deposited in the Sequence Read Archive database (accession number SRP106500; https://trace.ncbi.nlm.nih.gov/Traces/sra/?study=SRP106500).

## AUTHOR CONTRIBUTIONS

S.H.N. and L.W.S. designed the study. S.H.N. conducted the experiments, analyzed the data, and drafted the manuscript. Sequencing of 16S rRNA genes was performed in the TrEnD laboratory using workflows designed by M.S. and M.B. All authors contributed to data interpretation and manuscript revisions.
