# Supplementary material for: The influence of diet and environment on the gut microbial community of field crickets
Source: Ecol Evol. 2018 Apr 16;8(9):4704–20. doi: 10.1002/ece3.3977 (PMC5938447; doi:10.1002/ece3.3977)
Supplement: Supplementary file 1 [file ECE3-8-4704-s001.pdf]

Supplementary Data

The influence of diet and environment on the gut microbial community of field crickets

Soon Hwee Ng, Michael Stat, Michael Bunce, Leigh W. Simmons

Supplementary Methods

Commands for analysing sequence data using the mothur software package

The mothur commands were adapted from the MiSeq standard operating procedure provided by the developers of the mothur software package[1-3].

mothur > summary.seqs(fasta=fastafile)
mothur > screen.seqs(fasta=current, group= groupsfile, summary=current, maxambig=0, minlength=200, maxlength=260)
mothur > unique.seqs()
mothur > count.seqs(name=current, group=current)
mothur > summary.seqs(count=current)
mothur > align.seqs(fasta=current, reference=silva.nr_v123.pcr.align, flip=t)
mothur > summary.seqs(fasta=current, count=current)
mothur > screen.seqs(fasta=current, count=current, summary=current, maxambig=0, minlength=240, maxlength=260, maxhomop=8)
mothur > summary.seqs(fasta=current, count=current)
mothur > filter.seqs(fasta=current, vertical=T, trump=.)
mothur > unique.seqs(fasta=current, count=current)
mothur > pre.cluster(fasta=current, count=current, diffs=2)
mothur > chimera.uchime(fasta=current, count=current, dereplicate=t)
mothur > remove.seqs(fasta=current, accnos=current, count=current)
mothur > summary.seqs(fasta=current, count=current)
mothur > classify.seqs(fasta=current, count=current, reference=gg_13_5_99.fasta, taxonomy=gg_13_5_99.gg.tax, cutoff=80)
mothur > remove.lineage(fasta=current, count=current, taxonomy=current, taxon=Chloroplast-Mitochondria-unknown-Archaea-Eukaryota-g__Wolbachia)
mothur > dist.seqs(fasta=current, cutoff=0.20)
mothur > cluster(column=current, count=current)
mothur > remove.rare(list=current, count=current, nseqs=1, label=0.03)
mothur > make.shared(list=current, count=current, label=0.03)
mothur > classify.otu(list=current, count=current, taxonomy=current, label=0.03)
mothur > rarefaction.single(shared=current, calc=sobs, freq=100)
mothur > summary.single(shared=current, calc=nseqs-coverage-sobs-invsimpson-chao, subsample=T, label=0.03)

References

1 Schloss, P. D. & Westcott, S. L. *MiSeq SOP*, <http://www.mothur.org/wiki/MiSeq_SOP> (2016).

2 Kozich, J. J., Westcott, S. L., Baxter, N. T., Highlander, S. K. & Schloss, P. D. Development of a dual-index sequencing strategy and curation pipeline for analyzing amplicon sequence data on the MiSeq Illumina sequencing platform. *Applied and Environmental Microbiology*, doi:10.1128/aem.01043-13 (2013).

3 Schloss, P. D. *et al.* Introducing mothur: open-source, platform-independent, community-supported software for describing and comparing microbial communities. *Appl Environ Microbiol* **75**, 7537-7541, doi:10.1128/AEM.01541-09 (2009).

Supplementary figures

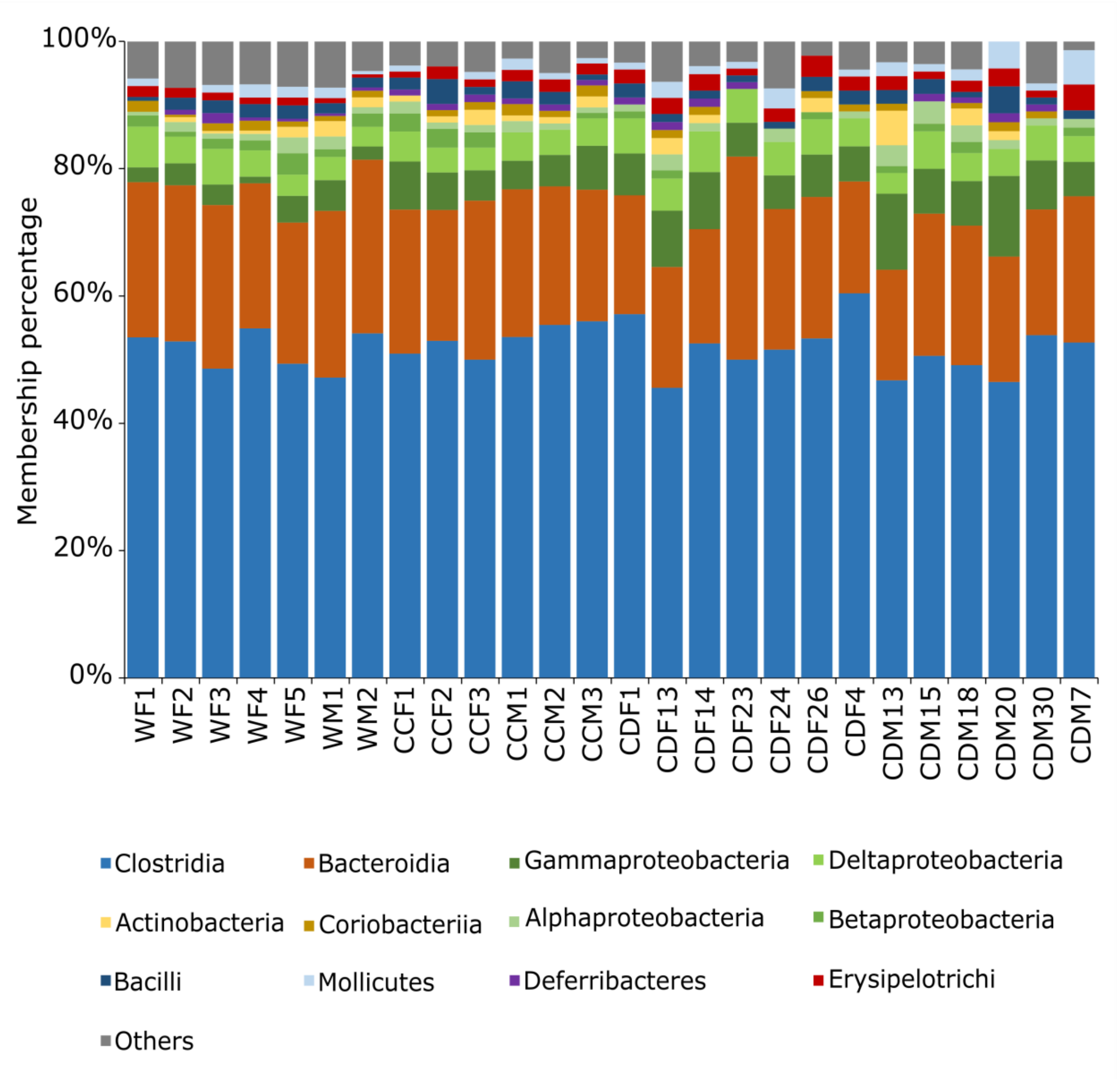

Supplementary Figure S1. Community proportional membership of bacterial Class across all samples. Taxa with less than 1% membership in cricket samples are grouped within Others. No significant difference was detected among diet groups (PERMANOVA; P = 0.102, R^2=0.127). WF, wild female cricket; WM, wild male cricket; CCF, cat chow female cricket; CCM, cat chow male cricket; CDF, chemically-defined diet female; cricket CDM, chemically-defined diet male cricket.

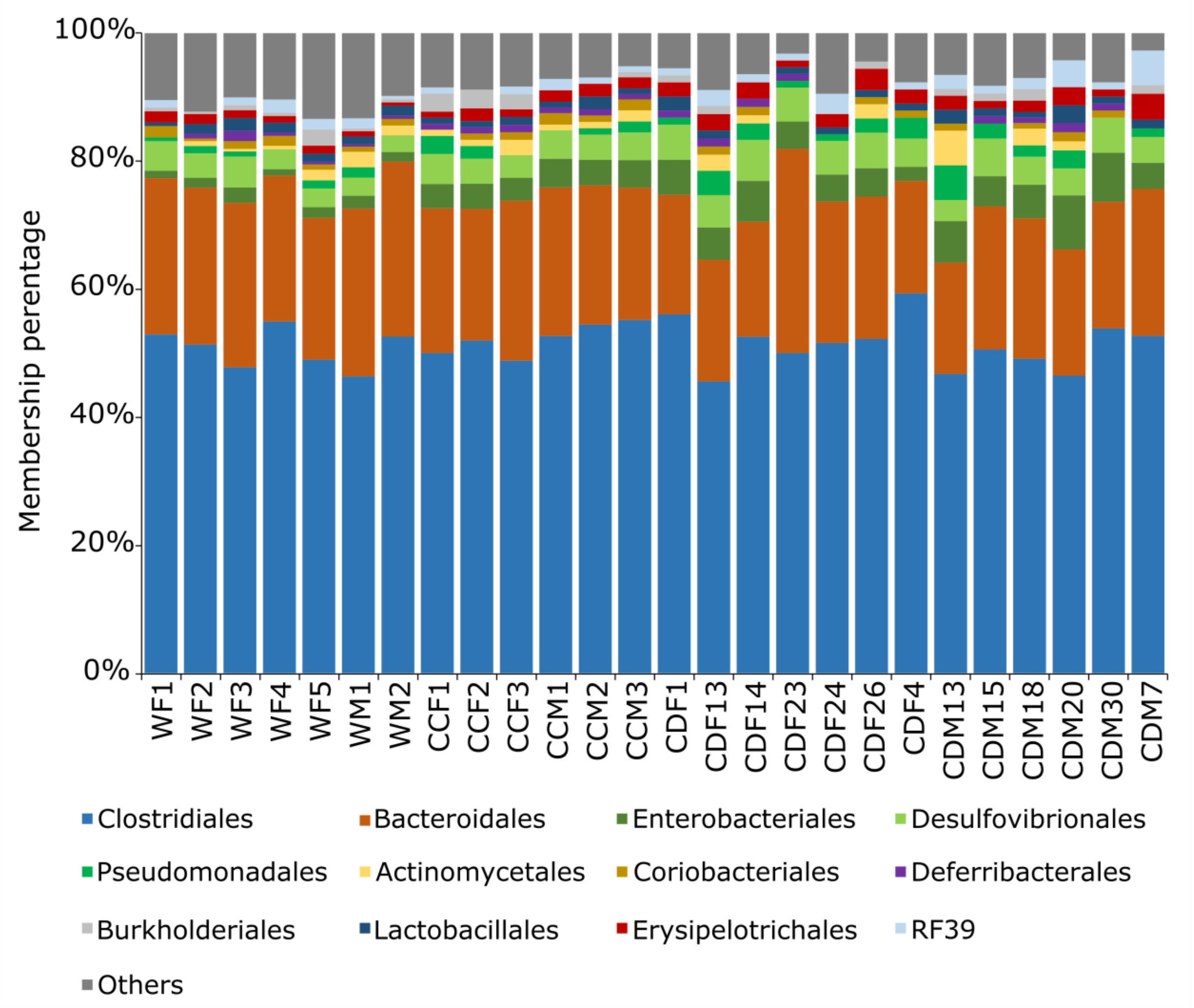

Supplementary Figure S2. Community proportional membership of bacterial Order across all samples. Taxa with less than 1% membership in cricket samples are grouped within Others. No significant difference was detected among diet groups (PERMANOVA; P = 0.078, R^2=0.136). WF, wild female cricket; WM, wild male cricket; CCF, cat chow female cricket; CCM, cat chow male cricket; CDF, chemically-defined diet female; cricket CDM, chemically-defined diet male cricket.

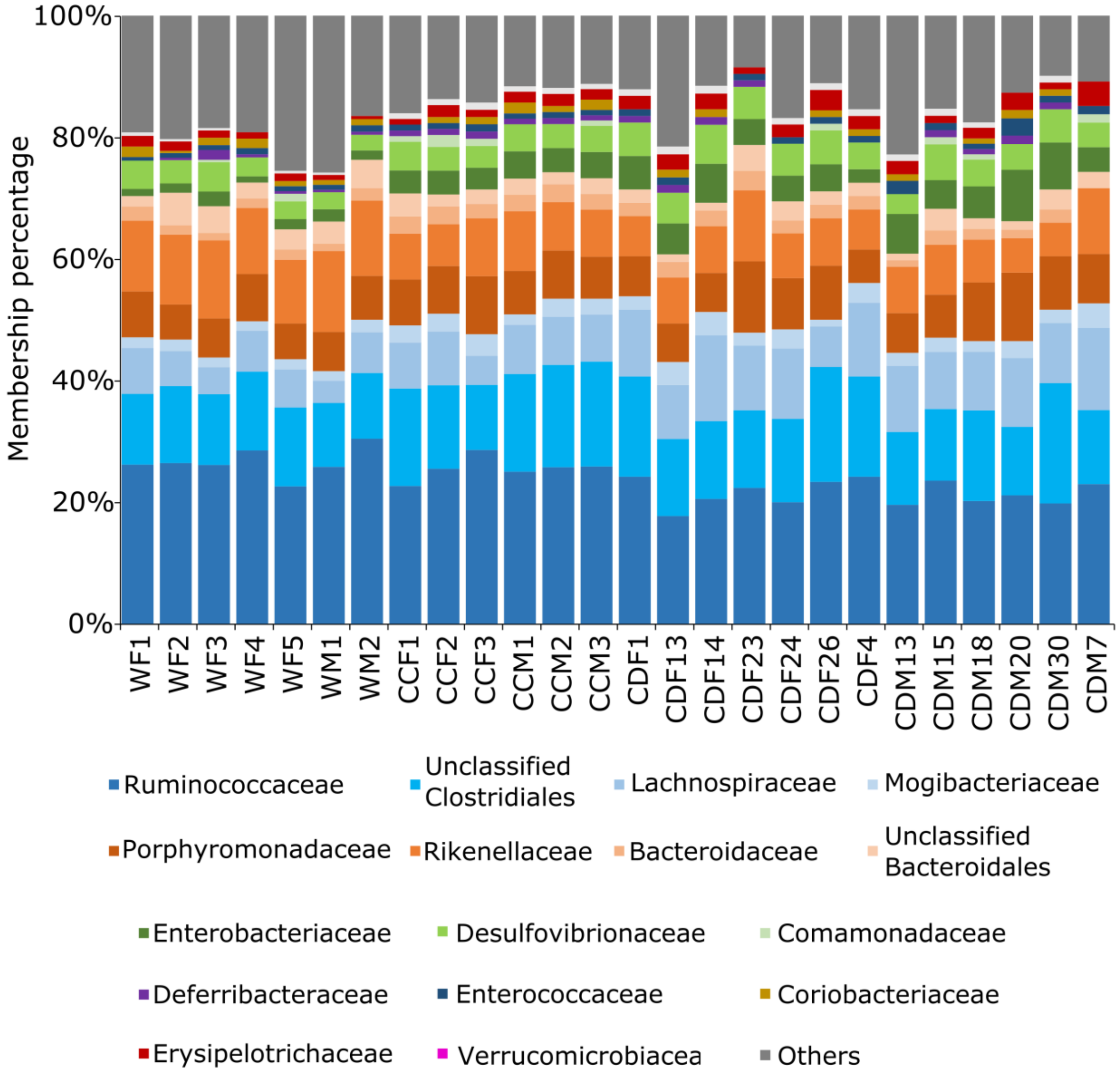

Supplementary Figure S3. Community proportional membership of bacterial Family across all samples. Taxa with less than 1% membership in cricket samples are grouped within Others. No significant difference was detected among diet groups (PERMANOVA; P = 0.210, R^2=0.106). WF, wild female cricket; WM, wild male cricket; CCF, cat chow female cricket; CCM, cat chow male cricket; CDF, chemically-defined diet female; cricket CDM, chemically-defined diet male cricket.

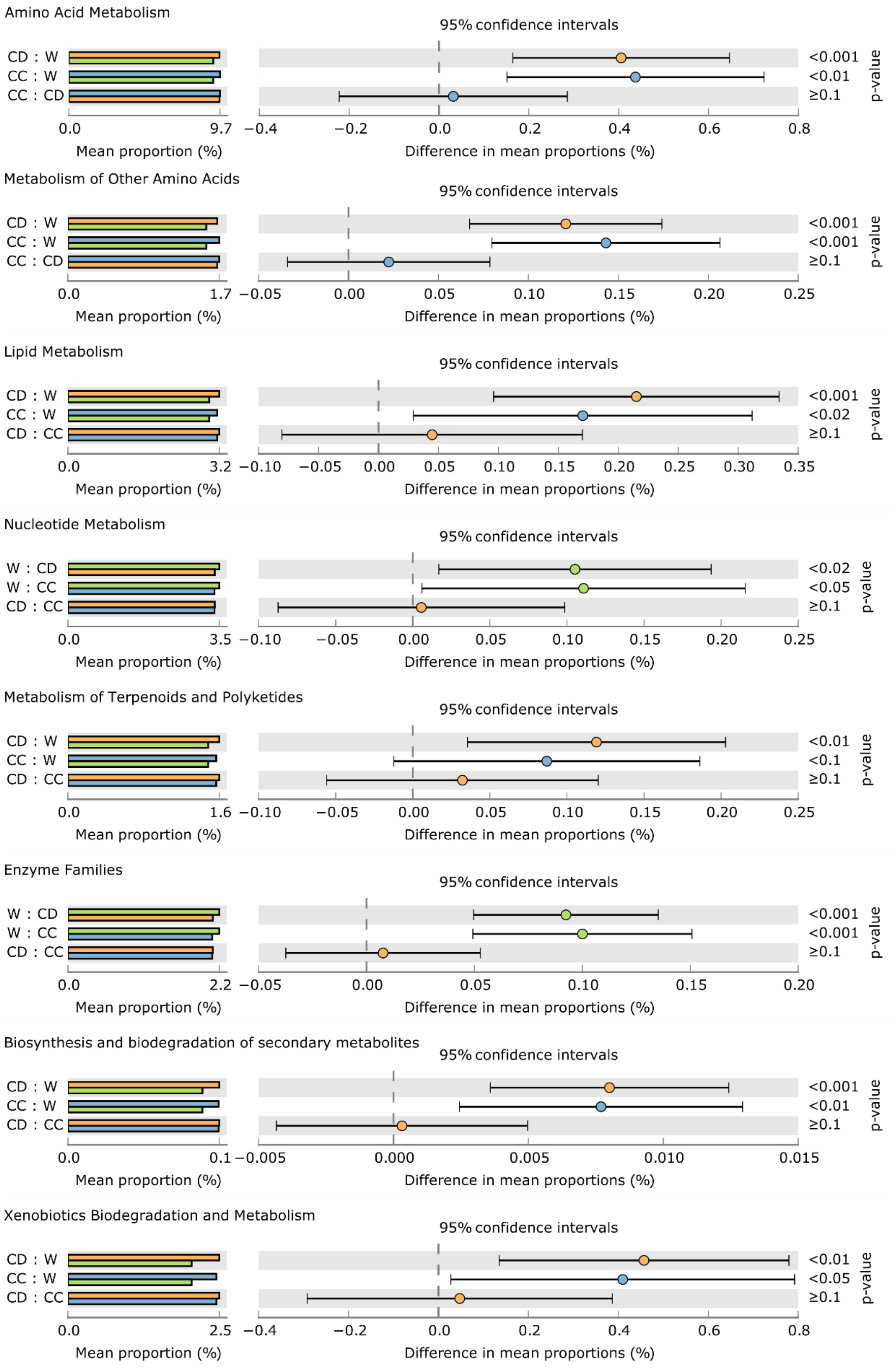

Supplementary Figure S4. Tukey-Kramer post-hoc tests of the eight Kyoto Encyclopedia of Genes and Genomes (KEGG)-Level 2 categories. Wild (W) crickets were significantly different from both chemically-defined diet (CD) crickets and cat chow (CC) crickets in all categories.

Supplementary tables

Supplementary Table S1. Relative abundance of bacteria taxa in the gut of 3 cricket groups: wild crickets, and lab-reared crickets fed either cat chow (CC) or chemically-defined diets (CD). Values are mean relative abundance with standard deviation.

Supplementary Table S2. Operational taxonomic units (OTUs) that are differentially represented in the 3 cricket groups (CD, chemically-defined diet; CC, cat chow; wild) identified by linear discriminant analysis effect size (LEfSe). Number of discriminative features with abs LDA score > 2 : 147.

Supplementary Table S3. Statistics and relative abundance of each Kyoto Encyclopedia of Genes and Genomes (KEGG)-Level 3 metagenomics category for individual cricket sample.

Supplementary Table S1. Relative abundance of bacteria taxa in the gut of 3 cricket groups: wild crickets, and lab-reared crickets fed either cat chow (CC) or chemically-defined diets (CD). Values are mean relative abundance with standard deviation.

Bacterial taxon				Relative abundance (%)		
Phylum	Class	Order	Family	Wild	CC	CD
**Actinobacteria**				0.27 ± 0.27	0.93 ± 0.38	0.2 ± 0.5
	Actinobacteria	Actinomycetales		0.11 ± 0.23	0.9 ± 0.36	0.19 ± 0.49
			Brevibacteriaceae	0 ± 0	0 ± 0	0.02 ± 0.07
			Cellulomonadaceae	0.01 ± 0.01	0 ± 0	0 ± 0
			Corynebacteriaceae	0.09 ± 0.21	0.89 ± 0.36	0.15 ± 0.48
			Dermabacteraceae	0 ± 0	0 ± 0	0 ± 0.01
			Geodermatophilaceae	0 ± 0	0.01 ± 0.02	0 ± 0
			Microbacteriaceae	0 ± 0	0 ± 0	0.01 ± 0.02
			Micrococcaceae	0 ± 0.01	0 ± 0	0 ± 0
			Tsukamurellaceae	0 ± 0	0 ± 0	0.01 ± 0.02
			Actinomycetales_unclassified	0.01 ± 0.01	0 ± 0	0 ± 0
	Coriobacteriia	Coriobacteriales	Coriobacteriaceae	0.16 ± 0.1	0.03 ± 0.02	0.01 ± 0.01
**Bacteroidetes**				28.69 ± 15.78	42.66 ± 12.4	74.1 ± 10.57
	Bacteroidia	Bacteroidales		27.99 ± 14.87	40.64 ± 13.07	72.63 ± 12.49
			Bacteroidales_unclassified	4.83 ± 4.91	3.8 ± 0.59	5.05 ± 3.94
			Bacteroidaceae	1.43 ± 2.34	6.23 ± 7.18	0.27 ± 0.25
			Odoribacteraceae	0.24 ± 0.33	0 ± 0	0.06 ± 0.05
			Porphyromonadaceae	11.12 ± 5.76	20.56 ± 4.24	58.57 ± 15.73
			Rikenellaceae	9.74 ± 4.73	7.95 ± 4.82	8.66 ± 3.99
	Cytophagia	Cytophagales	Cytophagaceae	0 ± 0.01	0 ± 0	0 ± 0
	Flavobacteriia	Flavobacteriales	Weeksellaceae	0.65 ± 1.58	2.02 ± 2.37	1.46 ± 4.85
	Sphingobacteriia	Sphingobacteriales	Sphingobacteriaceae	0 ± 0.01	0 ± 0	0 ± 0.01
	Bacteroidetes_unclassified			0.03 ± 0.05	0 ± 0	0 ± 0

Bacterial taxon				Relative abundance (%)		
Phylum	Class	Order	Family	Wild	CC	CD
**Cyanobacteria**	4C0d-2	YS2	YS2_unclassified	0.01 ± 0.04	0 ± 0	0 ± 0
**Deferribacteres**	Deferribacteres	Deferribacterales		0.28 ± 0.37	0.13 ± 0.08	0.02 ± 0.03
			Deferribacteraceae	0.14 ± 0.19	0.13 ± 0.08	0.02 ± 0.03
			Deferribacterales_unclassified	0.14 ± 0.38	0 ± 0	0 ± 0
**Firmicutes**				53.79 ± 16.25	30.92 ± 5.63	16.34 ± 5.54
	Bacilli			1.33 ± 1.32	1.46 ± 1.22	0.52 ± 0.54
		Bacillales		0.01 ± 0.01	0.01 ± 0.01	0.05 ± 0.12
			Paenibacillaceae	0 ± 0	0.01 ± 0.01	0 ± 0
			Staphylococcaceae	0.01 ± 0.01	0 ± 0	0.05 ± 0.12
			Bacillales_unclassified	0 ± 0.01	0 ± 0	0 ± 0
		Gemellales	Gemellaceae	0 ± 0	0 ± 0.01	0 ± 0
		Lactobacillales		1.32 ± 1.32	1.44 ± 1.21	0.47 ± 0.54
			Enterococcaceae	0.86 ± 0.78	1.42 ± 1.24	0.47 ± 0.54
			Lactobacillaceae	0.01 ± 0.02	0 ± 0	0 ± 0
			Leuconostocaceae	0.02 ± 0.05	0 ± 0	0 ± 0
			Streptococcaceae	0.44 ± 0.62	0.02 ± 0.05	0 ± 0
	Clostridia			51.93 ± 16.52	28.62 ± 5.49	14.3 ± 4.29
		Clostridia_unclassified		0.08 ± 0.09	0.09 ± 0.05	0 ± 0.01
		Clostridiales		51.84 ± 16.55	28.54 ± 5.49	14.29 ± 4.29
			Clostridiales_unclassified	7.27 ± 1.94	8.96 ± 2.93	3.27 ± 1.75
			Christensenellaceae	0.73 ± 0.12	0 ± 0	0 ± 0
			Dehalobacteriaceae	5.32 ± 3.24	0.05 ± 0.09	0.33 ± 0.5
			Eubacteriaceae	0.05 ± 0.12	0 ± 0.01	0 ± 0.01
			Lachnospiraceae	1.42 ± 1.13	1.27 ± 0.42	3.12 ± 1.53
			Mogibacteriaceae	3.76 ± 2.08	1.66 ± 0.49	0.62 ± 0.6
			Ruminococcaceae	32.42 ± 14.04	16.54 ± 5.43	6.77 ± 2.53
			Veillonellaceae	0.62 ± 0.39	0 ± 0	0 ± 0.01

Bacterial taxon				Relative abundance (%)		
Phylum	Class	Order	Family	Wild	CC	CD
	Erysipelotrichi	Erysipelotrichales	Erysipelotrichaceae	0.52 ± 0.35	0.84 ± 1.04	1.52 ± 1.6
	Firmicutes_unclassified			0.01 ± 0.01	0 ± 0	0 ± 0
**Fusobacteria**	Fusobacteriia	Fusobacteriales	Fusobacteriaceae	2.08 ± 4.04	0 ± 0	0 ± 0
**Lentisphaerae**	Lentisphaeria	Victivallales	Victivallaceae	0 ± 0.01	0 ± 0	0 ± 0
**Planctomycetes**	vadinHA49	PeHg47	PeHg47_unclassified	0.46 ± 0.39	0 ± 0	0 ± 0
**Proteobacteria**				12.02 ± 3.11	23.35 ± 9.33	8.54 ± 5.39
	Alphaproteobacteria			0.6 ± 0.43	4.58 ± 3.68	0.18 ± 0.2
		Alphaproteobacteria_unclassified		0.16 ± 0.21	0 ± 0	0 ± 0
		Caulobacterales	Caulobacteraceae	0 ± 0.01	0 ± 0	0 ± 0
		RF32	RF32_unclassified	0.06 ± 0.13	0 ± 0	0 ± 0
		Rhizobiales		0.37 ± 0.39	4.57 ± 3.66	0.17 ± 0.19
			Beijerinckiaceae	0 ± 0.01	0 ± 0	0 ± 0
			Bradyrhizobiaceae	0.37 ± 0.39	4.57 ± 3.66	0.17 ± 0.19
			Rhizobiales_unclassified	0 ± 0	0 ± 0	0 ± 0
		Rhodobacterales	Rhodobacteraceae	0 ± 0	0 ± 0	0 ± 0
		Rhodospirillales	Acetobacteraceae	0 ± 0.01	0 ± 0	0 ± 0
		Sphingomonadales	Sphingomonadaceae	0 ± 0	0.01 ± 0.03	0 ± 0
	Betaproteobacteria			1.85 ± 1.58	0.05 ± 0.06	0.01 ± 0.01
		Betaproteobacteria_unclassified		0.65 ± 0.43	0 ± 0	0 ± 0
		Burkholderiales		0.54 ± 0.25	0.05 ± 0.06	0.01 ± 0.01

Bacterial taxon				Relative abundance (%)		
Phylum	Class	Order	Family	Wild	CC	CD
			Burkholderiaceae	0 ± 0	0.01 ± 0.02	0 ± 0
			Comamonadaceae	0.01 ± 0.02	0.02 ± 0.02	0 ± 0
			Oxalobacteraceae	0.53 ± 0.24	0.01 ± 0.03	0 ± 0
		Neisseriales	Neisseriaceae	0.62 ± 1.65	0 ± 0	0 ± 0
		Nitrosomonadales	Nitrosomonadales_unclassified	0 ± 0	0 ± 0	0 ± 0
		Rhodocyclales	Rhodocyclaceae	0.04 ± 0.1	0 ± 0	0 ± 0
	Deltaproteobacteria			5.38 ± 3.47	2.58 ± 0.74	1.52 ± 1.06
		Desulfarculales	Desulfarculaceae	0.64 ± 0.78	0 ± 0	0 ± 0
		Desulfobacterales	Desulfobacteraceae	0.57 ± 0.28	0 ± 0	0 ± 0
		Desulfovibrionales	Desulfovibrionaceae	4.17 ± 3.03	2.58 ± 0.74	1.52 ± 1.06
	Gammaproteobacteria			2.94 ± 1.72	16.15 ± 6.99	6.84 ± 4.79
		Enterobacteriales	Enterobacteriaceae	2.18 ± 1.7	10.22 ± 7.3	5.19 ± 4.66
		Pseudomonadales		0.31 ± 0.46	0.14 ± 0.29	1.65 ± 1.43
			Moraxellaceae	0.04 ± 0.06	0.02 ± 0.02	0.03 ± 0.06
			Pseudomonadaceae	0.27 ± 0.46	0.13 ± 0.29	1.63 ± 1.43
		Vibrionales	Vibrionaceae	0.03 ± 0.08	0 ± 0	0 ± 0
		Xanthomonadales	Xanthomonadaceae	0.41 ± 0.68	5.78 ± 8.77	0 ± 0
	Proteobacteria_unclassified			1.25 ± 1.13	0 ± 0	0 ± 0
**Synergistetes**	Synergistia	Synergistales	Synergistaceae	0.02 ± 0.04	0 ± 0	0 ± 0

Bacterial taxon				Relative abundance (%)		
Phylum	Class	Order	Family	Wild	CC	CD
**Tenericutes**				0.43 ± 0.38	1.14 ± 1.45	0.33 ± 0.42
	CK-1C4-19	CK-1C4-19_unclassified		0.09 ± 0.21	1.1 ± 1.42	0 ± 0.01
	Mollicutes	RF39	RF39_unclassified	0.34 ± 0.38	0.04 ± 0.04	0.33 ± 0.42
**Verrucomicrobia**	Verrucomicrobiae	Verrucomicrobiales	Verrucomicrobiaceae	0.89 ± 0.92	0.76 ± 0.41	0.35 ± 0.39
	Verrucomicrobia_unclassified			0 ± 0	0 ± 0	0.06 ± 0.19
**Bacteria_unclassified**				1.07 ± 0.41	0.1 ± 0.19	0.12 ± 0.16

Supplementary Table S2. Operational taxonomic units (OTUs) that are differentially represented in the 3 cricket groups (CD, chemically-defined diet; CC, cat chow; wild) identified by linear discriminant analysis effect size (LEfSe). Number of discriminative features with abs LDA score > 2 : 147.

OTU	Phylum	Family	Diet	LDA	P-value
**Otu0001**	Bacteroidetes	Porphyromonadaceae	CD	4.98958	0.001218
**Otu0002**	Bacteroidetes	Porphyromonadaceae	CD	4.92021	6.44E-05
**Otu0004**	Bacteroidetes	unclassified	CD	4.35453	0.000481
**Otu0007**	Bacteroidetes	Rikenellaceae	CD	4.3539	8.43E-05
**Otu0028**	Bacteroidetes	Rikenellaceae	CD	3.61872	0.00123
**Otu0016**	Firmicutes	Ruminococcaceae	CD	3.66399	0.012338
**Otu0023**	Firmicutes	Lachnospiraceae	CD	3.75375	0.00093
**Otu0024**	Firmicutes	unclassified	CD	3.72269	0.00063
**Otu0049**	Firmicutes	Lachnospiraceae	CD	3.59257	0.000202
**Otu0064**	Firmicutes	Erysipelotrichaceae	CD	3.49354	0.000455
**Otu0092**	Firmicutes	Lachnospiraceae	CD	3.24792	0.029361
**Otu0133**	Firmicutes	unclassified	CD	2.92228	0.029528
**Otu0191**	Firmicutes	Lachnospiraceae	CD	2.52639	0.038754
**Otu0017**	Proteobacteria	Pseudomonadaceae	CD	3.90379	0.001893
**Otu0061**	Proteobacteria	Desulfovibrionaceae	CD	3.32092	0.007179
**Otu0105**	Tenericutes	unclassified	CD	3.03923	0.000448
**Otu0074**	Actinobacteria	Corynebacteriaceae	CC	3.69152	0.001325
**Otu0003**	Bacteroidetes	Porphyromonadaceae	CC	4.48092	0.002863
**Otu0013**	Bacteroidetes	Porphyromonadaceae	CC	4.12204	0.016941
**Otu0020**	Bacteroidetes	Bacteroidaceae	CC	4.22167	0.036227
**Otu0022**	Bacteroidetes	Porphyromonadaceae	CC	3.98561	0.000434
**Otu0036**	Bacteroidetes	Rikenellaceae	CC	3.5474	0.040633

OTU	Phylum	Family	Diet	LDA	P-value
**Otu0038**	Bacteroidetes	Bacteroidaceae	CC	4.11131	0.002586
**Otu0051**	Bacteroidetes	Rikenellaceae	CC	3.85312	0.002587
**Otu0055**	Bacteroidetes	unclassified	CC	4.06253	4.52E-05
**Otu0063**	Bacteroidetes	Porphyromonadaceae	CC	3.90172	4.40E-05
**Otu0066**	Bacteroidetes	Rikenellaceae	CC	3.73886	0.000862
**Otu0069**	Bacteroidetes	unclassified	CC	3.11735	0.016213
**Otu0140**	Bacteroidetes	Rikenellaceae	CC	2.9589	0.011496
**Otu0169**	Deferribacteres	Deferribacteraceae	CC	2.89404	0.0006
**Otu0031**	Firmicutes	Ruminococcaceae	CC	3.94025	0.000177
**Otu0044**	Firmicutes	unclassified	CC	3.70634	0.000783
**Otu0048**	Firmicutes	unclassified	CC	3.99086	0.006452
**Otu0056**	Firmicutes	unclassified	CC	4.0061	9.83E-05
**Otu0068**	Firmicutes	unclassified	CC	3.45007	0.043225
**Otu0070**	Firmicutes	Ruminococcaceae	CC	3.74441	0.000297
**Otu0071**	Firmicutes	Ruminococcaceae	CC	3.41835	0.000529
**Otu0085**	Firmicutes	Ruminococcaceae	CC	3.2589	2.18E-05
**Otu0089**	Firmicutes	Ruminococcaceae	CC	3.48993	0.000206
**Otu0091**	Firmicutes	Ruminococcaceae	CC	3.63194	0.014175
**Otu0118**	Firmicutes	unclassified	CC	3.1726	0.003871
**Otu0123**	Firmicutes	unclassified	CC	2.69601	0.010487
**Otu0145**	Firmicutes	unclassified	CC	2.83959	0.006945
**Otu0212**	Firmicutes	Ruminococcaceae	CC	3.0291	0.000544
**Otu0264**	Firmicutes	unclassified	CC	2.83758	0.000548
**Otu0318**	Firmicutes	unclassified	CC	2.47517	0.018816
**Otu0010**	Proteobacteria	Enterobacteriaceae	CC	4.43384	0.008553

OTU	Phylum	Family	Diet	LDA	P-value
**Otu0025**	Proteobacteria	Enterobacteriaceae	CC	4.00758	0.003261
**Otu0030**	Proteobacteria	Bradyrhizobiaceae	CC	4.32302	0.002033
**Otu0052**	Proteobacteria	Xanthomonadaceae	CC	4.40974	0.027072
**Otu0086**	Proteobacteria	Desulfovibrionaceae	CC	3.24526	0.005125
**Otu0101**	Tenericutes	unclassified	CC	3.6948	0.001694
**Otu0021**	Bacteroidetes	Porphyromonadaceae	Wild	4.08019	7.59E-05
**Otu0042**	Bacteroidetes	unclassified	Wild	4.0839	0.000136
**Otu0053**	Bacteroidetes	Porphyromonadaceae	Wild	3.83959	4.75E-05
**Otu0079**	Bacteroidetes	Rikenellaceae	Wild	3.60284	5.48E-06
**Otu0097**	Bacteroidetes	Porphyromonadaceae	Wild	3.53449	5.52E-06
**Otu0111**	Bacteroidetes	unclassified	Wild	3.34205	0.000349
**Otu0115**	Bacteroidetes	Odoribacteraceae	Wild	3.2257	0.02181
**Otu0116**	Bacteroidetes	Rikenellaceae	Wild	3.39651	0.002209
**Otu0117**	Bacteroidetes	Rikenellaceae	Wild	3.36087	0.002209
**Otu0134**	Bacteroidetes	Rikenellaceae	Wild	3.32069	0.002209
**Otu0149**	Bacteroidetes	Porphyromonadaceae	Wild	3.08059	0.001272
**Otu0150**	Bacteroidetes	Bacteroidaceae	Wild	3.07988	4.63E-05
**Otu0172**	Bacteroidetes	Rikenellaceae	Wild	2.97341	0.000345
**Otu0173**	Bacteroidetes	Rikenellaceae	Wild	3.01792	0.002197
**Otu0186**	Bacteroidetes	Rikenellaceae	Wild	3.03737	0.000347
**Otu0196**	Bacteroidetes	Porphyromonadaceae	Wild	2.83818	0.002197
**Otu0203**	Bacteroidetes	Rikenellaceae	Wild	2.84693	0.000349
**Otu0206**	Bacteroidetes	Porphyromonadaceae	Wild	2.80818	0.002209
**Otu0207**	Bacteroidetes	unclassified	Wild	2.73006	0.000341
**Otu0243**	Bacteroidetes	Rikenellaceae	Wild	2.77085	0.002197

OTU	Phylum	Family	Diet	LDA	P-value
**Otu0245**	Bacteroidetes	Rikenellaceae	Wild	2.79545	0.002197
**Otu0274**	Bacteroidetes	unclassified	Wild	2.81027	0.000341
**Otu0006**	Firmicutes	Ruminococcaceae	Wild	4.33272	0.000229
**Otu0008**	Firmicutes	Ruminococcaceae	Wild	4.22947	0.004105
**Otu0011**	Firmicutes	Ruminococcaceae	Wild	4.13909	0.001714
**Otu0012**	Firmicutes	Dehalobacteriaceae	Wild	4.37918	0.000298
**Otu0015**	Firmicutes	Mogibacteriaceae	Wild	3.98792	0.003377
**Otu0019**	Firmicutes	Ruminococcaceae	Wild	3.83455	0.012265
**Otu0046**	Firmicutes	Ruminococcaceae	Wild	3.75771	6.59E-05
**Otu0047**	Firmicutes	Ruminococcaceae	Wild	3.51503	0.005449
**Otu0054**	Firmicutes	Mogibacteriaceae	Wild	3.43408	0.039683
**Otu0072**	Firmicutes	Ruminococcaceae	Wild	3.60083	5.48E-06
**Otu0075**	Firmicutes	Ruminococcaceae	Wild	3.29981	0.00753
**Otu0077**	Firmicutes	Lachnospiraceae	Wild	3.47183	0.010046
**Otu0090**	Firmicutes	unclassified	Wild	3.46164	4.72E-05
**Otu0093**	Firmicutes	Ruminococcaceae	Wild	3.47098	0.000347
**Otu0095**	Firmicutes	Ruminococcaceae	Wild	3.27288	0.017817
**Otu0098**	Firmicutes	unclassified	Wild	3.39327	5.52E-06
**Otu0099**	Firmicutes	unclassified	Wild	3.38193	0.000349
**Otu0100**	Firmicutes	Veillonellaceae	Wild	3.41474	5.52E-06
**Otu0103**	Firmicutes	Ruminococcaceae	Wild	3.399	0.000349
**Otu0104**	Firmicutes	unclassified	Wild	3.2738	2.46E-05
**Otu0108**	Firmicutes	Streptococcaceae	Wild	3.41312	0.00131
**Otu0113**	Firmicutes	Ruminococcaceae	Wild	3.44058	0.000136
**Otu0114**	Firmicutes	unclassified	Wild	3.36107	0.000348

OTU	Phylum	Family	Diet	LDA	P-value
**Otu0120**	Firmicutes	Ruminococcaceae	Wild	3.00427	0.000623
**Otu0121**	Firmicutes	unclassified	Wild	3.23782	0.029072
**Otu0122**	Firmicutes	unclassified	Wild	3.29283	4.72E-05
**Otu0124**	Firmicutes	Ruminococcaceae	Wild	3.33582	5.48E-06
**Otu0125**	Firmicutes	Christensenellaceae	Wild	3.19268	4.72E-05
**Otu0130**	Firmicutes	unclassified	Wild	3.30317	0.002209
**Otu0135**	Firmicutes	Ruminococcaceae	Wild	3.16558	5.48E-06
**Otu0139**	Firmicutes	Ruminococcaceae	Wild	2.96124	0.009201
**Otu0141**	Firmicutes	Ruminococcaceae	Wild	3.09847	4.72E-05
**Otu0146**	Firmicutes	Erysipelotrichaceae	Wild	2.95305	0.002197
**Otu0151**	Firmicutes	Ruminococcaceae	Wild	2.95658	0.012637
**Otu0157**	Firmicutes	unclassified	Wild	2.99396	0.001745
**Otu0160**	Firmicutes	Ruminococcaceae	Wild	3.03804	0.002209
**Otu0162**	Firmicutes	Ruminococcaceae	Wild	2.91074	0.025754
**Otu0164**	Firmicutes	Mogibacteriaceae	Wild	2.79442	0.027454
**Otu0165**	Firmicutes	Enterococcaceae	Wild	3.04356	0.000349
**Otu0176**	Firmicutes	Ruminococcaceae	Wild	2.99554	0.002209
**Otu0179**	Firmicutes	unclassified	Wild	2.95952	0.002209
**Otu0184**	Firmicutes	Ruminococcaceae	Wild	2.79406	0.000345
**Otu0198**	Firmicutes	Ruminococcaceae	Wild	2.92591	0.002209
**Otu0201**	Firmicutes	unclassified	Wild	2.80278	0.000349
**Otu0205**	Firmicutes	Ruminococcaceae	Wild	2.77921	0.002209
**Otu0215**	Firmicutes	Ruminococcaceae	Wild	2.78705	0.002209
**Otu0218**	Firmicutes	Christensenellaceae	Wild	2.87672	0.000347
**Otu0221**	Firmicutes	Ruminococcaceae	Wild	2.79564	4.69E-05

OTU	Phylum	Family	Diet	LDA	P-value
**Otu0222**	Firmicutes	Ruminococcaceae	Wild	2.81837	0.002209
**Otu0225**	Firmicutes	unclassified	Wild	2.72989	0.002209
**Otu0229**	Firmicutes	Ruminococcaceae	Wild	2.8272	0.002209
**Otu0235**	Firmicutes	Ruminococcaceae	Wild	2.8928	4.69E-05
**Otu0240**	Firmicutes	Mogibacteriaceae	Wild	2.79573	0.002185
**Otu0246**	Firmicutes	Ruminococcaceae	Wild	2.8698	0.000345
**Otu0339**	Firmicutes	Christensenellaceae	Wild	2.79556	0.002197
**Otu0035**	Fusobacteria	Fusobacteriaceae	Wild	4.02423	0.000349
**Otu0147**	Planctomycetes	unclassified	Wild	3.08093	0.002209
**Otu0167**	Planctomycetes	unclassified	Wild	3.16202	0.000349
**Otu0219**	Planctomycetes	unclassified	Wild	2.85776	4.69E-05
**Otu0033**	Proteobacteria	Desulfovibrionaceae	Wild	3.93417	0.001805
**Otu0059**	Proteobacteria	unclassified	Wild	3.75644	5.52E-06
**Otu0081**	Proteobacteria	unclassified	Wild	3.62253	5.52E-06
**Otu0088**	Proteobacteria	Desulfobacteraceae	Wild	3.48003	5.52E-06
**Otu0094**	Proteobacteria	Oxalobacteraceae	Wild	3.50387	5.48E-06
**Otu0110**	Proteobacteria	Desulfovibrionaceae	Wild	3.32098	4.75E-05
**Otu0177**	Proteobacteria	unclassified	Wild	3.10852	4.63E-05
**Otu0220**	Proteobacteria	unclassified	Wild	2.69605	0.002197
**Otu0242**	Proteobacteria	Desulfovibrionaceae	Wild	2.76584	0.002197
**Otu0277**	Proteobacteria	Desulfovibrionaceae	Wild	2.77379	0.002209
**Otu0155**	Tenericutes	unclassified	Wild	3.06966	0.002209
**Otu0128**	unclassified	unclassified	Wild	3.25121	0.000347
**Otu0129**	unclassified	unclassified	Wild	2.97128	0.047235
**Otu0255**	unclassified	unclassified	Wild	2.7946	0.002197
